# Population-wide DNA methylation polymorphisms at single-nucleotide resolution in 207 cotton accessions reveal epigenomic contributions to complex traits

**DOI:** 10.1038/s41422-024-01027-x

**Published:** 2024-10-17

**Authors:** Ting Zhao, Xueying Guan, Yan Hu, Ziqian Zhang, Han Yang, Xiaowen Shi, Jin Han, Huan Mei, Luyao Wang, Lei Shao, Hongyu Wu, Qianqian Chen, Yongyan Zhao, Jiaying Pan, Yupeng Hao, Zeyu Dong, Xuan Long, Qian Deng, Shengjun Zhao, Mengke Zhang, Yumeng Zhu, Xiaowei Ma, Zequan Chen, Yayuan Deng, Zhanfeng Si, Xin Li, Tianzhen Zhang, Fei Gu, Xiaofeng Gu, Lei Fang

**Affiliations:** 1 Zhejiang Provincial Key Laboratory of Crop Genetic Resources, the Advance Seed Institute, Key Laboratory of Plant FactoryGeneration-adding Breeding, Ministry of Agriculture and Rural Affairs, College of Agriculture and Biotechnology, Zhejiang University, Hangzhou, Zhejiang China; 2 Damo Academy, Alibaba Group, Hangzhou, Zhejiang China; 3 Hainan Institute of Zhejiang University, Yazhou Bay Science and Technology City, Yazhou District, Sanya, Hainan China; 4 Hupan Lab, Hangzhou, Zhejiang China; 5 Biotechnology Research Institute, Chinese Academy of Agricultural Sciences, Beijing, China

**Keywords:** Plant molecular biology, DNA methylation

## Abstract

DNA methylation plays multiple regulatory roles in crop development. However, the relationships of methylation polymorphisms with genetic polymorphisms, gene expression, and phenotypic variation in natural crop populations remain largely unknown. Here, we surveyed high-quality methylomes, transcriptomes, and genomes obtained from the 20-days-post-anthesis (DPA) cotton fibers of 207 accessions and extended the classical framework of population genetics to epigenetics. Over 287 million single methylation polymorphisms (SMPs) were identified, 100 times more than the number of single nucleotide polymorphisms (SNPs). These SMPs were significantly enriched in intragenic regions while depleted in transposable elements. Association analysis further identified a total of 5,426,782 *cis-*methylation quantitative trait loci (*cis*-meQTLs), 5078 *cis*-expression quantitative trait methylation (*cis*-eQTMs), and 9157 expression quantitative trait loci (eQTLs). Notably, 36.39% of *cis*-eQTM genes were not associated with genetic variation, indicating that a large number of SMPs associated with gene expression variation are independent of SNPs. In addition, out of the 1715 epigenetic loci associated with yield and fiber quality traits, only 36 (2.10%) were shared with genome-wide association study (GWAS) loci. The construction of multi-omics regulatory networks revealed 43 *cis*-eQTM genes potentially involved in fiber development, which cannot be identified by GWAS alone. Among these genes, the role of one encoding CBL-interacting protein kinase 10 in fiber length regulation was successfully validated through gene editing. Taken together, our findings prove that DNA methylation data can serve as an additional resource for breeding purposes and can offer opportunities to enhance and expedite the crop improvement process.

## Introduction

Phenotypic variation arises from the integrative impacts of genetic and epigenetic variations, along with environmental dynamics. While significant progress has been made in understanding the genome and genetic variations through genome-wide association studies (GWAS) in recent decades,^1,2^ the role of epigenomic modifications in shaping phenotypic diversity in crops remains largely unexplored.

DNA methylation is one of the most well-studied epigenetic marks since the 1950s.^3^ The addition of a methyl group at the C-5 position of cytosine residues plays a key role in many biological processes across a wide range of organisms, from bacteria to humans, including suppressing the activity of transposable elements (TEs),^4^ maintaining genome stability,^5^ regulating gene expression,^6^ and affecting the binding of proteins.^7^ In plants, DNA methylation of cytosine bases occurs in all cytosine sequence contexts: the symmetric CG and CHG contexts (in which H = A, T, or C) and the asymmetric CHH context.^8,9^ CG methylation is propagated by the DNA methylation maintenance system during DNA replication, whereas non-CG (CHG and CHH) methylation is sustained by a self-reinforcing loop mechanism.^10–13^ DNA methylation is known to regulate several important agronomic traits such as flowering time,^14,15^ seed dormancy,^14^ yield,^16,17^ fruit ripening,^18^ and crop resilience.^19,20^ Also, the semi-dwarf trait in wheat and rice, which plays a significant role in the success of the Green Revolution, is controlled by epigenomic mechanisms.^21^ However, it is still unclear which type of DNA methylation contributes more significantly to regulating complex traits in plants.

High-throughput profiling of the epigenome at the cellular level has the potential to uncover a hidden layer of gene expression regulation. Pioneering population-level epigenetic studies have been conducted in animal^22^ and plant genomes, such as in *Arabidopsis thaliana*,^23,24^ maize,^25,26^ rice,^27^ and soybean.^24^ These studies have demonstrated that epi-mutations accumulate over evolutionary timescales and are associated with adaptation to ecologically diverse environments.^23,28^ The formation of agronomic traits is coordinated by a complex interplay of genetic, epigenetic, and environmental factors. Investigating whether population-wide variation in DNA modifications contributes to improving crop traits is a promising avenue for further research.^29^

Cotton is a crucial natural fiber crop, serving as a sustainable resource for the global textile industry. The fibers are developed through a highly synchronized differentiation process of cells originating from the seed coat. The quality of fiber is determined during the secondary cell wall thickening stage, which usually begins around 20 days post anthesis (DPA).^30^ Throughout the development and maturation of the fibers, dynamic DNA methylation patterns have been observed,^31,32^ creating an opportunity to investigate inter‐accession epigenomic variation and its association with fiber traits. Here, we report a comprehensive population-wide analysis that integrates methylome, transcriptome, and genome data collected from 20-DPA fibers of 207 cotton accessions. Through this analysis, we aim to identify key genes and epigenetic regulatory loci that play a role in shaping fiber traits. Our findings provide a genome-wide repository of DNA methylation modifications associated with lint yield and fiber quality traits. This resource will aid in advancing the breeding efforts of upland cotton by enabling genomic and epigenomic selection strategies for trait enhancement.

## Results

### Construction and characterization of DNA methylation variation map

**Fig. 1:**
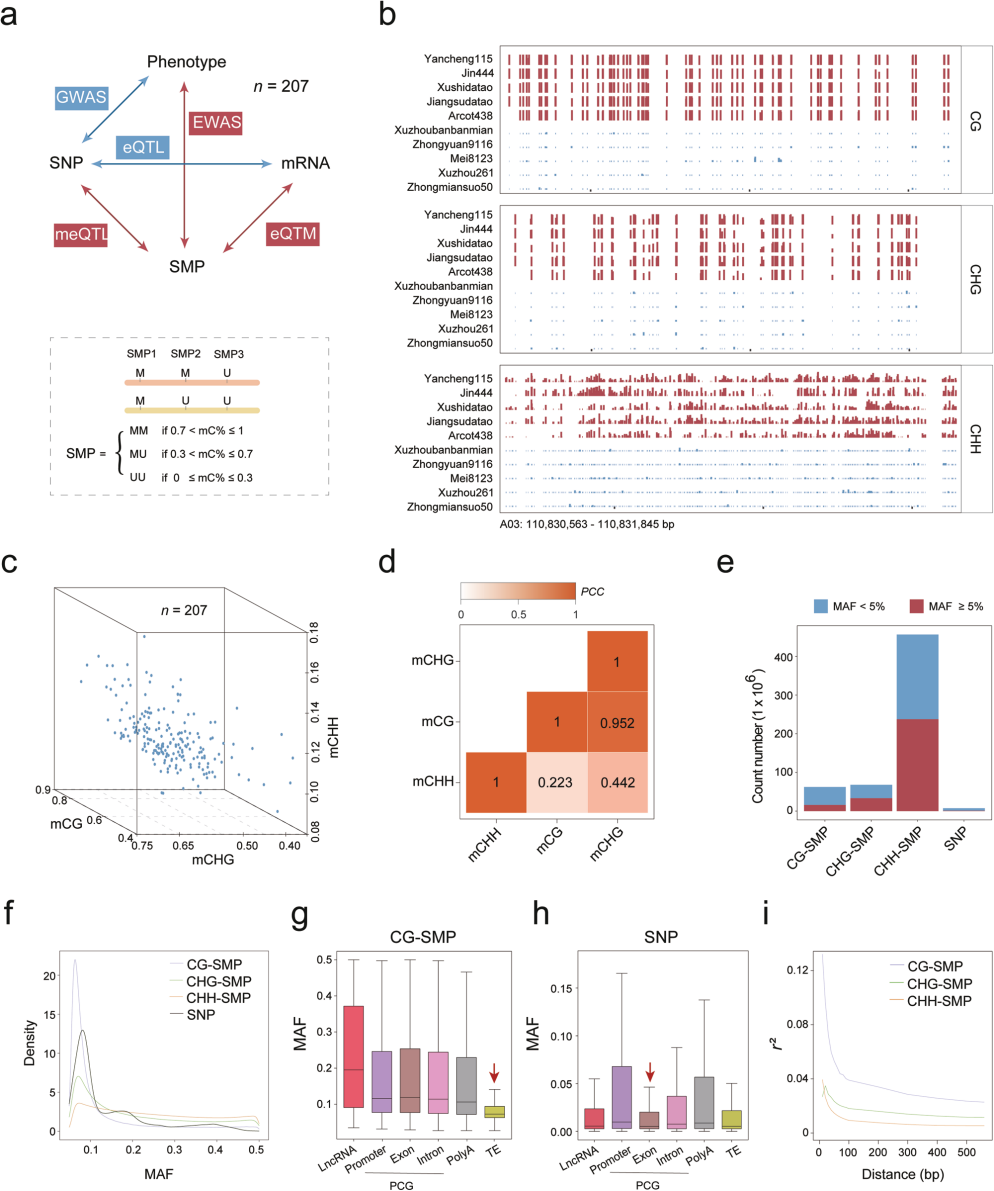
Extensive variation pattern of DNA methylation in a natural population. **a** Workflow of the multiple-omics association. The bottom panel refers to the definition of SMP. **b** An example of genomic regions exhibiting DNA methylation diversity among different accessions. Each track represents a distinct accession. **c** 3D plot illustrating the diversity of DNA methylation among different accessions. **d** The correlation among three different DNA methylation contexts. **e** Bar plot demonstrating the number and portion of SMP with MAF greater than 0.5. **f** Density plot showing MAF distributions for CG, CHG, CHH-SMPs and SNPs. **g**, **h** Box plots showing the distribution of MAFs of SMP (**g**) and SNP (**h**) for CG sites across different genomic features. **i** Comparison of LD decay among different DNA methylation contexts (vertical axis: LD level; horizontal axis: pairwise distance).

A core germplasm upland cotton population (CUCP1)^1,33^ with 207 accessions was employed for this multi-omics integrative study (Fig. 1a). All plants were grown in Hangzhou, China in 2021, and 20-DPA fibers at the secondary cell wall (SCW) thickening stage were harvested for whole-genome bisulfite sequencing (WGBS), and transcriptome sequencing (RNA-seq) in parallel. Samples for high-quality genome, methylome, and transcriptome analyses were obtained for all accessions. WGBS and RNA-seq generated 54 billion and 4.42 billion clean reads, respectively, for a total of 17.76 trillion base pairs (Supplementary information, Fig. S1a, b and Tables S1, S2). Methylome reads were mapped against the upland cotton reference genome TM-1 version2.1 (v2.1),^34^ achieving an average mapping rate of 74.90% ± 3.55%. All sequenced methylomes had an average coverage depth exceeding 15 folds (Supplementary information, Table S1). After strict data processing and quality control, 62.32 million (M), 66.06 M, and 433.01 M methylated cytosines were quantified in CG, CHG, and CHH contexts, respectively (Supplementary information, Fig. S1c‒e and Table S1). The RNA-seq profiling was conducted using two biological replications for each accession. The Pearson correlation coefficients (PCCs) of paired biological replicate transcriptomes were significantly higher than those of randomly selected samples (Wilcox test, *P* < 2.2 × 10^‒16^), confirming the high quality of our data (Supplementary information, Fig. S1f). In parallel, 3.05 trillion-base pair whole-genome sequencing (WGS) of the accessions generated 1,282,390 biallelic high-quality SNPs (minor allele frequency (MAF) > 0.05 and missing ratio < 20%), which were used for expression quantitative trait loci (eQTL) and expression quantitative trait methylation (eQTM) mapping (Fig. 1a). The collected datasets provide a comprehensive study of accession-specific gene expression and DNA methylation status in upland cotton, enabling an investigation into the influence of DNA methylation on agronomic traits.

Methylome data generated from the 207 accessions showed that the cotton genome is highly methylated, especially in heterochromatin regions (Supplementary information, Fig. S2). The genome-wide DNA methylation was about 72%, 55%, and 11% in contexts of CG, CHG, and CHH sites, respectively (Supplementary information, Table S1). The CG methylation ratio in our study is consistent with previous estimates in cotton,^14^ higher than that in orange fruit (CG: 41%),^18^ while lower than that in barley (CG: 94.7%).^35^ The 207 accessions also exhibited genome-wide variation in cytosine methylation (CG-interquartile range (IQR) = 8.08%; CHG-IQR = 6.75%, and CHH-IQR = 1.37%) (Fig. 1b, c). Genome-wide CG DNA methylation exhibited a strong correlation with CHG DNA methylation levels (PCC = 0.95, *P* < 2.2 × 10^‒16^), while a low correlation with CHH DNA methylation levels (PCC = 0.22, *P* = 0.0012) (Fig. 1d). Within each accession, the genome-wide distribution of DNA methylation followed a binomial distribution (Supplementary information, Fig. S3), reflecting that each site is either completely methylated or unmethylated. This characteristic justifies the conversion of methylation levels (%) at each cytosine to binary values to represent methylation variation, the feasibility of which has been validated in a previous human study.^36^ Therefore, the definition of a single methylation polymorphism (SMP) was adopted as the DNA methylation variation on each allele of homologous chromosomes at a specific cytosine location. Three epi-alleles can be identified for an SMP: both methylated (MM allele, 70% < mC% ≤ 100%), both unmethylated (UU allele, 0 ≤ mC% ≤ 30%), and heterozygosis (MU allele, 30% < mC% ≤ 70%) (Fig. 1a).

Phylogenetic analysis based on SMPs grouped the 207 accessions into four clades (Supplementary information, Fig. S4). Clade II included American landraces Stoneville 2B (STV2B) and 86-1, and modern cultivars developed from STV2B collected from the Chinese Yellow River cotton-growing area. Clade III contained American landrace Deltapine 15 (DPL15), and cultivars developed primarily from DPL15 planted in all three cotton-growing areas of China (Supplementary information, Fig. S4).

The number of common SMPs (MAF ≥ 0.05, 16.15 M for CG, 33.41 M for CHG, and 237.74 M for CHH) in the cotton genome is much larger than the number of SNPs (1.28 M) (Fig. 1e). The MAF of CHH-SMPs is 0.22, larger than the values obtained for CHG-SMPs (MAF = 0.11), CG-SMPs (MAF = 0.05) and SNPs (MAF = 0.14) (Fig. 1f).

MAF values of SMPs vary across different genomic features (Fig. 1g; Supplementary information, Fig. S5a and Table S3). The CG-SMP MAF value of TEs is half of that of protein-coding genes (PCGs) that include exons and promoters (Fig. 1g), while the MAF value of SNPs was similar among TEs and PCGs (Fig. 1h). In the cotton genome, many repetitive sequences are located in exon regions. CG-SMPs located within exons can be classified as either TEs or non-TEs. The MAF of CG-SMPs located within TEs was significantly lower than those not in repetitive sequences (Wilcox test, *P* = 6.8 × 10^‒4^) (Supplementary information, Fig. S5b). It was interesting to note that the CG-SMP MAF was significantly lower in TEs, although TEs are usually highly methylated. Notably, common CG-SMPs (MAF ≥ 0.05) were three times more enriched in intragenic regions compared to other SMP types (CG-SMP: 27.53%, CHG-SMP: 9.78%, and CHH-SMP: 10.49%) (Supplementary information, Fig. S5c). This result is consistent with a previous report in *Arabidopsis*, demonstrating that varied genic methylation tends to occur in the CG context within the transcribed region.^37^

To characterize the relationships between adjacent DNA methylation loci,^38^ the concept of linkage disequilibrium (LD) was applied to DNA methylation, henceforth termed methylation disequilibrium (MD). The average distance at which MD decayed to half of its maximum value was about 50 bp (Fig. 1i), consistent with previous estimations in humans and *Arabidopsis*.^22,36^ Notably, the decay of MD was significantly faster than LD (Supplementary information, Fig. S5d), which was reported to span over 300 kb in the same population.^1^ In addition, the MD of CHH was lower than those of CHG and CG (Fig. 1i), suggesting that methylation at the symmetrical CG and CHG sequences might be preferentially maintained across mitotic and meiotic cell divisions.^39^ Thus, DNA methylation is an important source of variation in intragenic regions.

### Genetic variations in gene-enriched regions have major impacts on the methylome

**Fig. 2:**
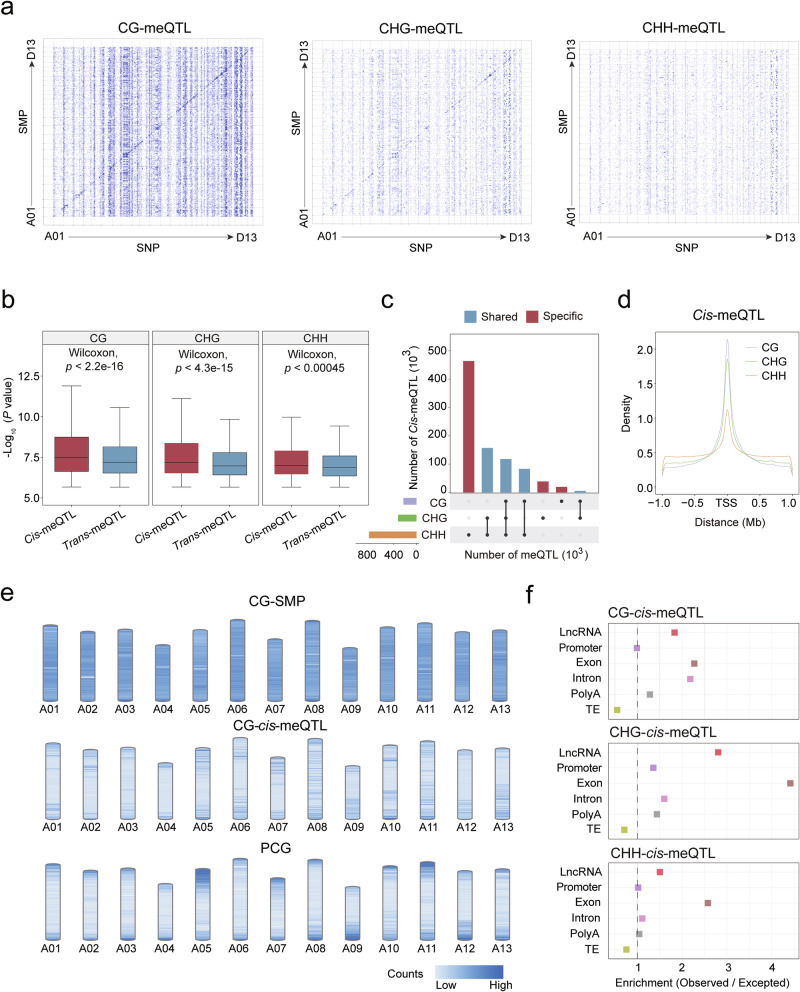
The genetic basis of three contexts of DNA methylation. **a** The genomic distribution of SMP and their associated SNPs. The *x*-axis indicates the genomic positions of the significant SNPs, while the *y*-axis shows the genomic positions of the corresponding SMPs. 50,000 SMPs of CG, CHG and CHH were chosen for genome-wide meQTL analysis. **b** Box plot showing the distribution of ‒log_10_(*P*) of *cis*- and *tran*s-meQTL. Boxes show the medians and IQRs. **c** UpsetR plots illustrating the proportions of shared *cis*-meQTLs among different DNA methylation contexts (Fisher’s exact test, ***P < 0.001). **d** The distance between DMR and the significant SNP. **e** The genomic distribution of *cis*-meQTL across the genome. **f** Enrichment and depletion of *cis*-meQTLs across different genomic features.

To characterize the genetic impacts on DNA methylation, we mapped the genetic variants that affect DNA methylation. First, a genome-wide random sampling of 50,000 CG-SMPs, CHG-SMPs, and CHH-SMPs, accounting for 0.31%, 0.15%, and 0.021% of each SMP type, respectively, was selected to reassess meQTL in both *cis*- and *trans*-effects, in parallel. We define meQTL as *cis-*meQTL if the distance between the SNP and the associated SMP is within 1 Mb. 119,685, 37,831, and 24,683 meQTLs were identified in CG, CHG, and CHH contexts, respectively. Although a large number of *trans*-meQTLs were identified through the meQTL analysis (Fig. 2a), *cis*-meQTLs exhibited greater levels of significance compared to *trans*-meQTLs (Fig. 2b).

To minimize false positives and reduce the computational burden, only *cis*-meQTLs were chosen for further analysis. In parallel, all SMPs (*n* = 287.30 M) were subjected to *cis*-meQTL analysis via the software fastQTL.^40^ In total, 5,426,782 *cis*-meQTLs were identified including 940,794 CG-*cis*-meQTLs, 883,280 CHG-*cis*-meQTLs, and 3,602,708 CHH-*cis*-meQTLs. Only a small proportion of DNA methylation loci (5.82%, 2.64%, and 1.52% of CG, CHG, and CHH sites, respectively) were found to be involved in *cis*-meQTLs. A proportion of *cis*-meQTLs of three DNA methylation contexts (CG, CHG, and CHH) showed co-localization (Fig. 2c). Additionally, the distance between the SNP and its associated SMP of CG-*cis*-meQTLs exhibited shorter spans in comparison to those observed in the CHG and CHH contexts (Fig. 2d).

The distribution of *cis*-meQTL is uneven across the genome, showing a higher density near the chromosome ends (Fig. 2e; Supplementary information, Fig. S6). To assess the pattern of *cis*-meQTL enrichment in different genomic features, we explored distribution bias using Fisher’s exact test, comparing the observed frequency with the expected frequency. The results showed that *cis*-meQTLs were significantly enriched in intragenic regions (Fisher’s exact test, *P* < 2.2 × 10^‒16^), but significantly depleted in TEs (Fisher’s exact test, *P* < 2.2 × 10^‒16^) (Fig. 2f).

### The involvement of SMPs in expression regulation

**Fig. 3:**
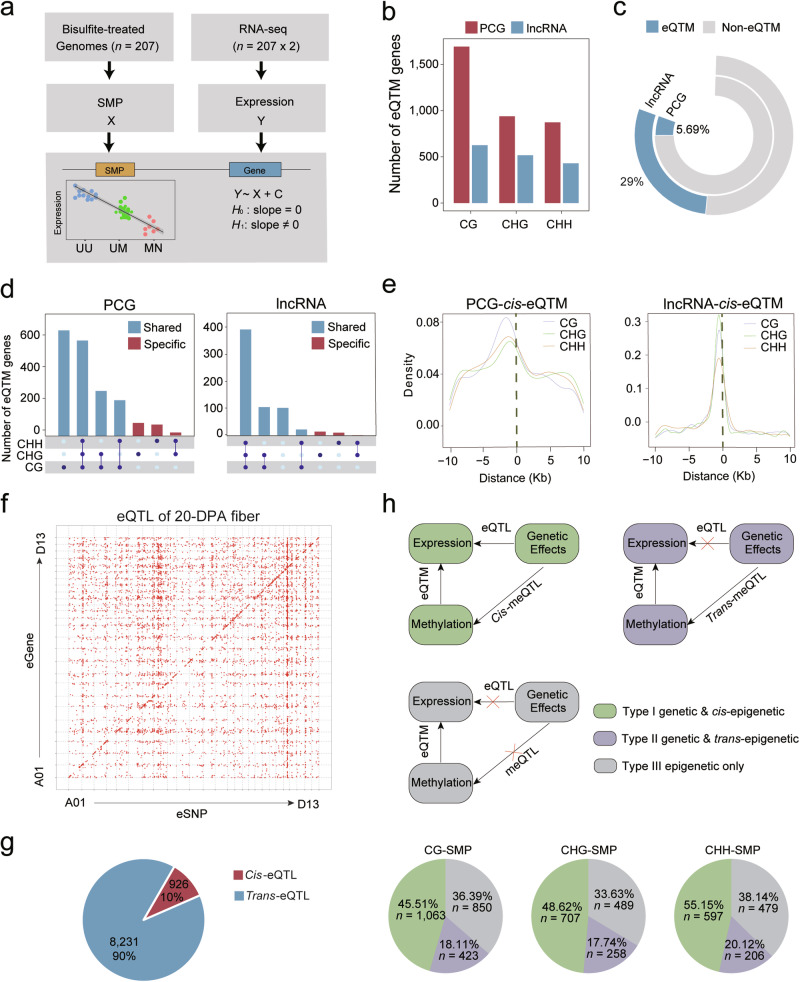
Gene expression variations influenced by DNA methylation. **a** Workflow of the eQTL analysis. **b** Number of *cis*-eQTMs identified in PCGs and lncRNAs. **c** Percentage of PCGs and lncRNAs influenced by DNA methylation. **d** UpsetR plots of overlapping and specific *cis*-eQTM genes. Right: PCGs; left: lncRNAs. *Cis*-eQTMs related to CG are labeled in red; others are painted in blue. **e** Distance of lead SMPs to the associated transcription start site. Left: PCGs; right: lncRNAs. **f** Scatter plot of high-confidence eSNP-expression associations. Each dot represents a detected eQTL. Expression of eGenes is plotted on the *y*-axis and eSNPs on the *x*-axis. **g** Pie plot showing the number of *cis*- and *trans*-eQTLs. **h** Characterization of eQTM genes identified in both eQTM and meQTL analyses. These loci were categorized into three groups. Genetic & *cis*-epigenetic regulated (type I), genetic & *trans*-epigenetic regulated (type II) and epigenetic regulated only (type III).

Given that *cis*-meQTLs are enriched in PCGs across natural populations (Fig. 2e, f), exploring the relationship between DNA methylation and gene expression holds significance. Thus, we investigated the impact of DNA methylation on local gene expression through eQTM analysis using transcriptomes from the same harvested tissues (Fig. 3a; Supplementary information, Table S2). The population-wide transcriptome analysis was performed against the reference genome of TM-1 v2.0 annotated with 71,994 PCGs.^34^ In total, 21,181 long noncoding RNAs (lncRNAs) were annotated in this study. 41,632 PCGs and 5469 lncRNAs expressed in more than 5% of the population were retrieved for determining eQTL and eQTM. A total of 5078 *cis*-eQTMs were identified via fastQTL software,^40^ consisting of 3505 PCG-eQTMs and 1573 lncRNA-eQTMs (Fig. 3b). They were mapped to 2619 genes, representing 5.69% of the PCGs and 29% of the lncRNAs expressed in 20-DPA fiber (Fig. 3c; Supplementary information, Table S4). The *cis*-eQTMs genes showed enrichment in processes including long-chain fatty acid metabolism, trichome branching, and glucose homeostasis, likely related to fiber development by Gene Ontology (GO) analysis (Supplementary information, Table S5). In addition, it is common to observe simultaneous association of *cis*-eQTMs genes among different methylation contexts (Fig. 3d). For example, *cis*-eQTMs genes associated with all three methylation contexts account for a large portion of all *cis*-eQTMs genes (30.85% for PCGs and 60.24% for lncRNAs) (Fig. 3d). The analysis revealed that the majority of eQTM genes were associated with CG methylation, comprising 91% and 96% of eQTM PCGs and lncRNAs, respectively (Fig. 3d). This indicates that CG methylation plays a more crucial role in gene regulation compared to the other two types of methylation. At the population level, 90% of *cis*-eQTMs were biased to positions upstream of PCGs and lncRNAs (Fig. 3e). Furthermore, we observed that methylation levels of CG-eQTMs and CHG-eQTM located in the proximal promoter were negatively correlated with gene expression compared to eQTMs located in distal gene regions and gene bodies (Supplementary information, Fig. S7).

eQTL mapping was subsequently performed by Efficient Mixed Model Analysis Expedited (EMMAX) using the obtained SNPs and expression profiles. A total of 9157 eQTLs were detected, involving 5921 eSNPs and 7398 eGenes (PCG, *n* = 5197; lncRNA, *n* = 1014) (Fig. 3f; Supplementary information, Table S6). They were further subdivided into 926 *cis*-eQTLs and 8231 *trans*-eQTLs according to relative eGene (genes regulated by eQTL) location using an empirical distance threshold (1 Mb) (Fig. 3g).^41,42^ A set of 67 genes encoding critical proteins in DNA methylation establishment were further investigated, from which we identified *cis*-eQTL and *cis*-eQTM for *De Novo 2* (*IDN2*), a gene involved in RNA-directed DNA methylation (Supplementary information, Fig. S8a and Table S7).

We adopted a strategy similar to that of Meng et al.,^43^ to cluster the patterns of genomic regulation for eQTM genes into three categories (Fig. 3h), genetic/*cis*-epigenetic regulated (type I), genetic/*trans*-epigenetic regulated (type II), and epigenetic regulated only (type III). Regarding the SMP of eQTM, we carried out meQTL analysis. The eQTM genes from type II constituted a small portion (less than 20%) of the total eQTM genes (Fig. 3h). The eQTM genes characterized as type III account for 33.63%‒38.14% of the share (Fig. 3h), indicating an active role of DNA methylation involved in gene expression regulation. The co-regulated genes showed enrichment in organonitrogen compound biosynthetic process (Fisher’s exact test, *P* = 3.6 × 10^‒8^), sulfur compound biosynthetic process (Fisher’s exact test, *P* = 5.29 × 10^‒7^), and acetyl-CoA biosynthetic process (Fisher’s exact test, *P* = 1.62 × 10^‒4^) (Supplementary information, Fig. S8a‒c).

### Epigenome-wide association studies for agronomic traits revealed a large number of elite epi-alleles

**Fig. 4:**
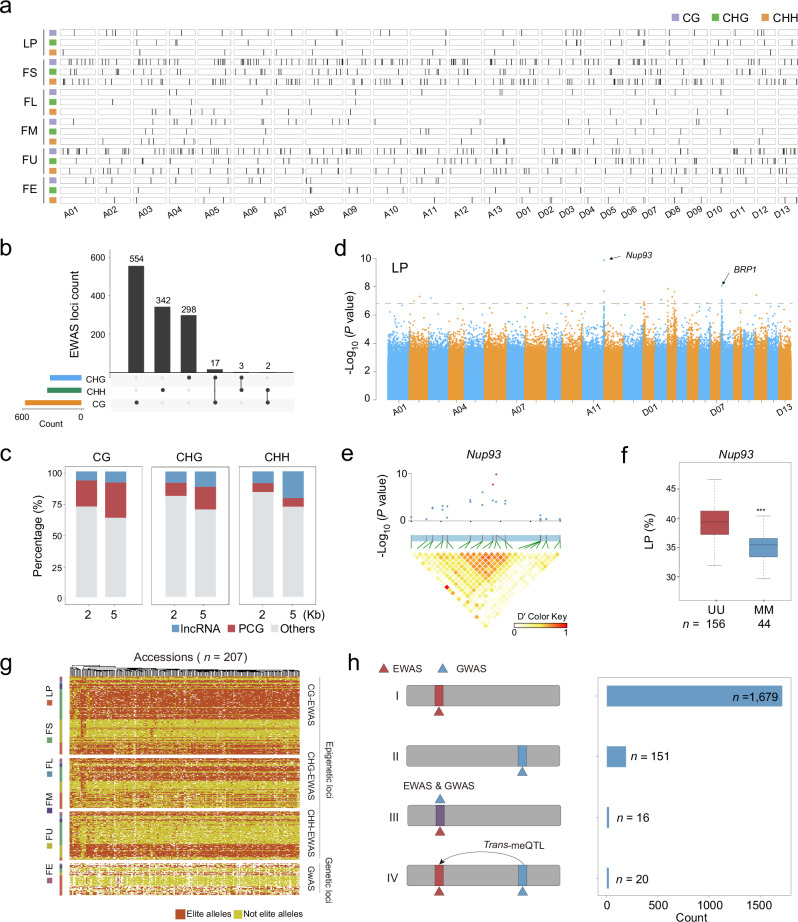
EWAS locus distribution and accumulative effects on agronomic traits. **a** Distribution of EWAS loci associated with agronomic traits. Fiber yield traits included lint percentage (LP); fiber quality: fiber length (FL), strength (FS), elongation (FE), micronaire (FM), and uniformity (FU). The loci associated with each were indicated by black vertical lines in the chromosome map. **b** UpsetR plot illustrating the overlap between CG-EWAS, CHG-EWAS, and CHH-EWAS. **c** Proportions of EWAS loci having a flanking gene within less than 2-kb and 5-kb regions. **d** Manhattan plot for the LP trait from EWAS analysis. The red arrow indicates the signal in Chr. A11. **e** Zoomed-in plot showing that the lead SMP represents the EWAS locus for LP on Chr. A11 and the signal coordinates are in the same methylation disequilibrium block. **f** LP of different epi-alleles for the locus shown in **e** (Student’s *t*-test, ***P < 0.001). **g** Heatmap showing haplotype distribution in the natural population according to CG-, CHG-, and CHH-EWAS loci, and also GWAS loci. Elite alleles are indicated in red. Each column represents an accession, and each row refers to a locus in the genome. **h** Characterization of loci identified in both EWAS and GWAS. These loci were categorized into four groups. Epigenetic regulated only (type I), genetic regulated only (type II), genetic/*cis*-epigenetic regulated (type III), and genetic/*trans*-epigenetic regulated (type IV).

Our *cis*-meQTL analysis revealed that the majority of SMPs were not associated with genetic variations, consistent with a previous study reporting that DNA methylation variation in *Arabidopsis* occurs independently of genetic variation.^22^ This suggests that epigenetic associations may not be captured by SNP-based markers. Using common SMPs (MAF ≥ 0.05) across the genome, instead of SNPs, we performed an epigenome-wide association study (EWAS) for nine traits using EMMAX software,^44^ which yielded 848 CG-EWAS loci (*P* = 6.52 × 10^‒8^), 467 CHG-EWAS loci (*P* = 3.09 × 10^‒8^), and 400 CHH-EWAS loci (*P* = 4.42 × 10^‒9^) (1715 in total) (Fig. 4a; Supplementary information, Table S8). Of these loci, 1010 were associated with yield-related traits, and 705 with fiber qualities (Supplementary information, Fig. S9a, b). When considering different contexts, the majority of EWAS loci were independent of each other, except for the 22 loci shared by at least two sequence contexts (Fig. 4b).

Approximately 27.67% of CG-EWAS loci, 19.92% of CHG-EWAS loci, and 16.19% of CHH-EWAS loci were located within a 2-kb flanking region of a protein-coding or lncRNA gene (Fig. 4c). Figure 4d and e present an example of an EWAS signal associated with the yield trait (lint percentage, LP) that occurred in the promoter of a gene encoding a nucleoporin interacting component (*Nup93*). Further, different epi-alleles corresponded to varying LP values (Two-tail unpaired Student’s *t*-test, *P* < 2.2 × 10^‒16^) (Fig. 4f).

To analyze the relationship between the genetic and epigenetic variance in trait variation, we constructed a map that combines EWAS loci with GWAS loci across all 207 accessions (Fig. 4g). GWAS identified 187 loci associated with nine traits related to fiber quality and yield.^1^ EWAS further identified a total of 1715 trait-associated epigenetic loci, of which only 16 (0.93%) were located near GWAS loci (< 20 kb) (Supplementary information, Table S8). For example, the epi-allele of the EWAS locus on chromosome A11 was significantly associated with LP, but no GWAS signal was detected at that locus (Supplementary information, Fig. S9c). Representative examples of EWAS loci that overlap with GWAS loci are shown in Supplementary information, Fig. S9d, e. In sum, these results illustrate that DNA methylation provides an additional layer of regulation to agronomic traits. Further, in our analysis of the EWAS loci that did not coincide with GWAS loci, we identified 992 loci with *trans*-meQTL effects, out of which 20 were associated with GWAS loci (Fig. 4h; Supplementary information, Table S8).

To assess the pyramiding effects of elite epi-alleles of EWAS loci for each trait of interest in the *Gossypium* *hirsutum* (*G.* *hirsutum*) germplasm, we compared traits among accessions carrying multiple elite epi-allelic combinations. The result revealed that accessions with more elite alleles consistently exhibit better trait performance (Supplementary information, Fig. S10). Since SNPs and SMPs represent different types of molecular information potentially associated with the phenotypes, utilizing a combination of SNPs and SMPs, we can improve the predictive performance for agronomic traits related to fiber yield and quality (Supplementary information, Fig. S11).

### Identification of fiber-related genes through multi-omics association analysis

**Fig. 5:**
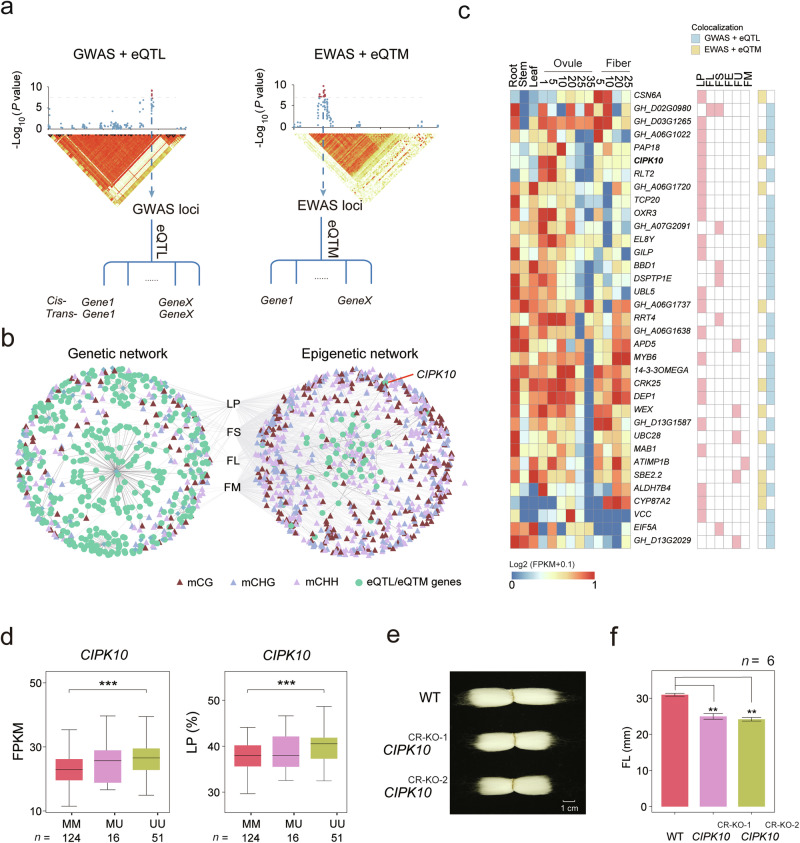
Genetic and epigenetic regulation networks associated with fiber development. **a** Analytical workflow for the construction of a functional GRN. Both eQTM and eQTL analyses were conducted to obtain causal sites in EWAS and GWAS loci, respectively. Loci within the same LD block (*r*^2^ > 0.1) were merged into one lead SNP, and eGenes within an LD block were clustered into a GRN. The same steps were also conducted for EWAS loci. **b** Gene networks regulating cotton fiber traits. Right: genetic variation-dependent network constructed by integrating GWAS and eQTL; left: epigenetic regulation network constructed by integrating EWAS and eQTM. **c** Heatmap showing candidate genes identified by colocation analysis. **d** Expression levels and LP of *CIPK10* across different epi-alleles. **e** Image illustrating the performance of gene editing (CRISPR knockout, *CR-KO*) on the eQTM gene *GhCIPK10*, which regulates fiber traits. **f** Fiber length in two *CIPK10*^*CR-KO*^ lines (Student’s *t*-test, **P < 0.01, *n* = 6).

Our multi-omics association analyses yielded 187 GWAS loci, 9157 eQTLs, 1715 EWAS loci, 5078 *cis*-eQTMs, and 5,426,782 *cis*-meQTLs. To examine the gene regulatory network (GRN) that complements the GWAS/EWAS loci, we constructed the GRN of gene expression by integrating the GWAS loci and eQTLs based on LD blocks (Fig. 5a).

51 GWAS loci were found to co-localize with 376 eQTLs within the same LD block (*r*^*2*^ > 0.1). The corresponding GRN for six fiber traits comprised 634 connections among 397 genes. Within this GRN, 77 (19.40%) eQTL genes were also eQTM genes, indicating co-regulation of gene expression by DNA methylation and genetic variation. Networks associated with four fiber traits (fiber yield (LP), strength (FS), length (FL), and micronaire (FM)) were depicted in Fig. 5b, including multiple genes known to be involved in fiber elongation, such as genes encoding Expansion A4,^45^ cellulose-synthase-like (CSL),^46^ ACTIN1,^47^ TCP transcription factors,^48^ bHLH transcription factors, and uridine diphosphate (UDP)-glucose.

An epigenetic regulation network, referred to as the epigenetic GRN, was established by integrating EWAS loci and eQTMs (Fig. 5a). In addition, an alternative epigenetic GRN was constructed using 47 eQTMs that co-localized with EWAS loci (Fig. 5b; Supplementary information, Table S9). A comparison between these two networks revealed only four genes in common, encoding trypsin protein and RIBOSOMAL PROTEIN EL8Y, GH_A06G1022, and aldehyde dehydrogenase (Fig. 5c; Supplementary information, Table S9). The minimal overlap between the two networks demonstrated the complex regulatory mechanisms governing fiber traits.

An EWAS locus (A03:4217197) associated with LP was located in the promotor of *CIPK10* that encodes a CBL-interacting protein kinase (Supplementary information, Table S8), which is a candidate fiber development gene in a *Gossypium barbadense* population.^2^ We also identified it as an eQTM gene, with DNA methylation status at a CG-SMP (A03:4217260) associated with both *CIPK10* expression (Student’s *t*-test, *P* = 2.5 × 10^‒4^) and LP (Student’s *t*-test, *P* = 2.5 × 10^‒4^) (Fig. 5d). Knocking out *CIPK10* through CRISPR/Cas9 gene editing system^49^ (Supplementary information, Fig. S12) resulted in shorter FL (*CIPK10* CR^KO-1^, 25.0 ± 0.8 mm; *CIPK10* CR^KO-2^, 24.22 ± 0.5 mm) compared to wild type (31.00 ± 0.4 mm) (Student’s *t*-test, *CIPK10* CR^KO-1^, *P* = 3.72 × 10^‒4^; *CIPK10* CR^KO-2^, *P* = 5.06 × 10^‒5^) (Fig. 5e, f).

### Prediction of functional CG methylation based on DNA sequence using DeepFDML

Deciphering the functional impacts of regulatory elements poses a crucial challenge in functional genomic studies for advancing next-generation crop breeding strategies. Deep learning models have been applied to uncover functional patterns in genetic elements by integrating genomic sequences with molecular features such as non-coding region transcription^50^ and *cis-*elements within promoters.^51^ However, such an approach for predicting functional epi-modification loci has not yet been developed.

**Fig. 6:**
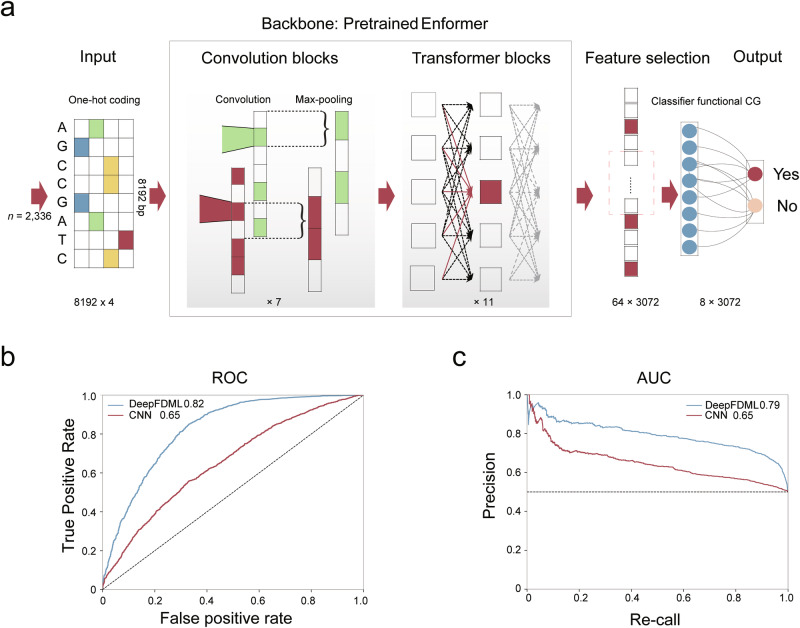
A convolutional neural network for functional CG site prediction. **a** Schematic diagram showing the pipeline of the proposed deep learning method. It mainly contains four components: input sequence, backbone, feature selection, and output layer. Each input was a one-hot-encoded DNA sequence of 8192 bp centered at the CG site. The backbone was from the pre-trained Enformer model. In feature selection, features of the middle eight positions were utilized. The output layer, a fully connected layer, was a binary classifier. **b** Receiver operating characteristic (ROC), curve measured on the whole dataset. **c** Precision-recall curve, measured on the whole dataset.

Here, we developed a deep learning model named Deep Functional DNA Methylation Loci (DeepFDML) to predict functional SMPs, which are SMPs associated with variations in gene expression. The DeepFDML model was trained on genomic sequences corresponding to functional CG sites, namely the 2336 non-redundant CG loci associated with 2423 CG-eQTMs (i.e., positive samples). To ensure the balance of training data, another set of 2336 CG-SMPs was randomly selected as the negative group. The flanking sequences of each CG-SMP locus were transformed via one-hot encoding (Fig. 6a).

To evaluate the impact of DNA methylation on gene expression, we first built a convolutional model consisting of a convolutional layer (kernel size of 11 and channel of 128) and a fully connected layer (Fig. 6a). The models were evaluated using a five-fold cross-validation approach, and the accuracy of our model reached 0.65 in both receiver operating characteristic curve (ROC) and the precision-recall curve (PRC) (Fig. 6b). Subsequently, a more complex DeepFDML model was constructed to improve the accuracy, adopting an architecture similar to the pre-trained Enformer model as its backbone.^52^ This advanced DeepFDML model contains a convolution part of seven convolution-pool blocks and a transformer part with 11 transformer encoding layers (Fig. 6a). The model achieved an ROC of 0.82 and an PRC of 0.78, significantly surpassing the performance of the convolutional model (Fig. 6b, c). Based on these results, we conclude that functional SMPs can be identified based on DNA sequence patterns through predictive models using deep learning approaches.

## Discussion

The investigation of DNA methylation’s impact on traits at a population level has been a hot topic for over a decade.^22,25,26,53^ Epigenetic recombinant inbred lines (epi-RILs) have been developed to analyze the effect of the epigenome on the phenotype,^15,54,55^ illustrating the relationship between phenotypic variation and phenotypic plasticity, independent of genetic factors. Population-wide DNA methylation studies in plants have been conducted in *A. thaliana*,^22,23^ maize,^25,26,53^ and soybean.^24^ Studies were also conducted in *A. thaliana* mutation accumulation (MA) lines to determine the rate at which single cytosines in the CG context acquire methylation, estimated at 2.56 × 10^−4^ per generation per haploid methylome.^56,57^ The intra-specific methylation variation seems to be broadly conserved.^39^ Short-term changes in DNA methylation are predominantly driven by spontaneous epi-permutational events.^28^ Thus, the phylogenetic tree based on cotton SMPs was consistent with accession pedigrees, in line with previous studies.^22,58–60^

We found that in the same cotton natural population, the number of DNA methylation polymorphisms is 100 times higher than that of genetic variation represented by SNPs (Fig. 1e). This finding is consistent with the rapid evolutionary pace of DNA methylation.^56,57^ Interestingly, the LD length is over 1000 times greater than MD (Fig. 1i). The complexity of DNA methylation haplotype in a given chromosomal region far exceeds that of SNPs. In our study, the average size of the MD block is 50 bp, a measurement consistent with previous reports on *Arabidopsis*^22^ and human genomes.^36^

One interesting discovery is that purifying selection of DNA methylation was observed in TE, which contrasts with the genetic variations featured by SNPs (Fig. 1g, h). The new pattern that CG DNA methylation variation biased to PCGs in the cotton population provides strong evidence that this epigenetic modification could regulate gene expression as an alternative source of DNA variation.

Population variation in DNA methylation is usually studied at the level of differentially methylated regions (DMRs) or average methylation levels of units.^23,25^ In mammals, the majority of research in this area has been conducted using SMP.^39^ One possible explanation is the cost associated with association analysis. The use of DMRs can significantly reduce the computational burden, but the definition and length of DMRs depend on the sample size and relevant parameters. To the best of our knowledge, the study presented here represents the first single nucleotide-level EWAS in the crop genome, performing association analysis at the individual base level.

In contrast to animals, plants exhibit three distinct contexts for DNA methylation (CG, CHG, and CHH). However, it remains unclear which type of DNA methylation plays a more significant role in regulating gene expression in plants. In this study, we selected 20-DPA fiber as the main focus of analysis in our study to examine the gene expression changes associated with genetic and epigenetic variations during the fiber-quality determining stage. The genome-wide catalogs of *cis*-regulatory eQTL, meQTL, and eQTM were well characterized. Because of the population-wide workload in this study, only one developmental stage of 20-DPA fiber was selected. QTLs that are specifically present in other tissues were not examined.

We found that DNA methylation regulated approximately 5.69% of PCGs and 29% of lncRNA (Fig. 3c). The majority of eQTM genes was associated with CG methylation (Fig. 3d), indicating that CG methylation plays a more important role in gene regulation. Similar results were previously observed in DNA methylation transferase null mutants in *Arabidopsis*.^61^ Further, an interesting discovery is that 36% of 2619 eQTM genes were not identified as eGenes of *cis*-eQTLs (Fig. 3h), indicating the existence of new types of sites with potential regulatory roles beyond genetic variation. Variations in CG methylation may contribute to missing heritability.

Epi-alleles contribute to agronomic traits. Studies using epi-RILs strongly indicate that epigenetics is involved in heritable phenotypic changes.^15,54^ Despite these studies, the EWAS focusing on an agronomic trait is rare. Our research identified 1715 epi-alleles that contribute to phenotypic variations. We observed the cumulative effects of elite EWAS alleles for each trait (Supplementary information, Fig. S10). The functional loci identified could serve as a valuable resource for understanding the regulatory mechanisms of complex traits. Interestingly, given that most of the EWAS and GWAS loci are independent of each other (Fig. 4h), we speculated that the EWAS loci may contribute to phenotypic variance in addition to genetic variations.

Identifying functional DNA methylation sites in the genome is challenging. Our results suggest that systematic population sequencing is an effective strategy, albeit costly. Therefore, predictive models based on DNA sequences are crucial for functional locus characterization and will benefit studies of other closely related species that lack population-scale DNA methylation data. Through population-scale multi-omics association analysis, our study generated an essential training dataset and demonstrated the predictability of functional DNA methylation loci (Fig. 6). While our current findings are rudimentary, it is crucial to emphasize the importance of developing models capable of generalization in the future.

## Methods

### Plant materials and DNA sequencing

A set of core germplasms (*n* = 207) from different regions of China were obtained. Plants were grown in Hangzhou, Zhejiang province. Fiber (20-DPA) of each genotype was harvested with two biological replications for WGBS and RNA-seq. For WGBS, genomic DNA was extracted using the DNeasy Plant Kit (Qiagen, Valencia, CA, USA) from frozen and ground 10-rosette leaves, and harvested just before bolting. Genomic DNA (2 μg) was fragmented with a Covaris S2 (Covaris, Woburn, MA, USA) to 200 bp, followed by end repair (End-It, Epicenter) and the addition of a 3′-tailing buffer (NEB). Cytosine-methylated adapters provided by Bio Scientific (NEXTflexTM Bisulfite-Seq Barcodes-12) (Bio Scientific Corp, Austin, TX, USA) were ligated to the sonicated DNA at 16 °C for 16 h using T4 DNA ligase (New England Biolabs, Ipswich, MA, USA). The adapter-ligated DNA was then subjected to two rounds of purification with AMPure XP beads (Beckman Coulter Genomics, Danvers, MA, USA), followed by sodium bisulfite conversion of an aliquot (≤ 450 ng) using the MethylCode kit (Life Technologies, Carlsbad, CA, USA) following the manufacturer’s instructions. The bisulfite-converted, adapter-ligated DNA molecules were enriched by four cycles of PCR with the following reaction mixture: 20 μL of bisulfite-converted sample, 25 μL of Kapa HiFi Hotstart Uracil^+^ Ready mix (Kapa Biosystems, Woburn, MA, USA), and 5 μL TruSeq PCR Primer Mix (Illumina, San Diego, CA) (50 μL final volume). The thermocycling parameters were: 95 °C for 2 min, 98 °C for 30 s, then four cycles of 98 °C for 15 s, 60 °C for 30 s, and 72 °C for 4 min, ending with one 72 °C hold for 10 min. The reaction products were purified using AMPure XP beads according to the manufacturer’s directions. WGBS libraries were sequenced paired-end 150 bp using the Illumina HiSeq 2500 (Illumina, San Diego, CA, USA) instrument as per the manufacturer’s instructions. Each library was sequenced to obtain a volume of 254.43 ± 5.38 million reads.

### DNA methylation data normalization and quality control

The raw WGBS data were processed using fastp (v0.12.2) to control the quality of reads and to remove adapter contamination, low-quality bases, and bases artificially introduced during library construction.^62^ WGBS reads were mapped to the *G. hirsutum* (TM-1) genome^34^ using Bowtie2 (v1.2.2), and implemented in Bismark with parameters (--score_min L,0, -0.2 -X 1000 --no-mixed --no-discordant).^63,64^ The resultant average mapping rate was 74.90% ± 3.55% (Supplementary information, Table S1); thus, it was not necessary to construct a pseudo-reference to improve the mapping rate for this cultivated cotton population. The non-conversion rate (the rate at which unmethylated cytosines failed to be converted to uracil) was calculated based on reads mapping to the lambda genome; the average conversion rate so obtained was 99.70 ± 0.03, suggesting highly efficient bisulfite conversion (Supplementary information, Table S1).

Only reads mapped to unique genomic locations were retained and used for further analysis. After filtering duplicated reads, we extracted methylated cytosines using the Bismark methylation extractor (v0.19.0) and retained those having more than five mapped reads.^64^ The methylation level at each cytosine site was then determined as the number of reads supporting cytosine methylation divided by the total number of reads.^65^ Hence, the methylation level ranged from 0 (unmethylated) to 1 (methylated).

Two quality-control steps were performed to screen cytosine sites: (1) removal of sites with < 5 coverage and high missing rate (missing in > 30% of the samples), and (2) removal of methylation loci that failed methylation detection at which an SNP was present. We then annotated SMPs according to their overlap with the following genomic regions: Refseq gene bodies, promoter regions (2 kb upstream of a transcription start site), poly (A) regions (2 kb downstream of a transcription end site), and TEs.

### DNA methylation across the population

DNA methylation at each mC locus was measured as mC% = 100 × methylated reads/(methylated reads + unmethylated reads). DNA methylation levels were translated into epi-alleles as follows:

$${{{{\rm{Individual}}}}\; {{{\rm{epi}}}}}\mbox{-}{{{\rm{alleles}}}}=\left\{\begin{array}{c}{{{\rm{MM}}}},{{{\rm{if}}}}\,0.7 \, < \,{{{\rm{mC}}}}\le 1\,\\ {{{\rm{MU}}}},0.3 \, < \,{{{\rm{mC}}}}\le 0.7\\ {{{\rm{UU}}}},{{{\rm{if}}}}\,0\le {{{\rm{mC}}}}\le 0.3\end{array}\right.$$

For two SMP loci SMP1 and SMP2, we propose that SMP1 has two alleles M1 and U1, and SMP2 likewise has two alleles M2 and U2. The frequencies of the four SMP alleles are denoted as p_M1_, p_U1_, p_M2_, p_U2_. Methylation equilibrium (ME) is defined as the case where SMP1 and SMP2 are independent; that is, no association exists between SMP alleles at the two loci. Based on the principle of independence, MD and Mr^2^ can be described using the formula:

$${{{\rm{MD}}}}={p}_{{{\rm{M1M2}}}} - {p}_{{{\rm{M1}}}}{p}_{{{\rm{M2}}}}$$

$${{{{{\rm{MD}}}}\; {{{\rm{coefficient}}}}}\,{{{\rm{M}}}}{{{\rm{r}}}}}^{2}$$

$${{{{\rm{Mr}}}}}^{2}=\frac{\left({{{\rm{MD}}}}\right)^{2}}{PM1PU1PM2PU2}$$

The range of Mr2 is also between 0 and 1.

The MAFs of SMPs were estimated by analyzing the variant sites (MAF ≥ 0.05) using vcftools (v 0.1.16).^66^

### Measurement of the methylation level of a region

The methylation level of a region was calculated based on the weighted DNA methylation:

$${\sum}_{i=1}^{n}{Ci}\bigg/{\sum}_{i=1}^{n}{Ci}+{Ti}$$

where C is the number of reads supporting methylated cytosine, T is the reads supporting unmethylated cytosine, i is the position of the cytosine, and *n* is the total number of cytosine positions.

### DNA genotyping

Genotype data were obtained in our previously published study.^1^ WGS data were quality controlled using fastp (v0.12.2) with default parameters. Genome and annotation files of TM-1 v2.1^34^ were indexed using a BWA (v0.7.17-r1188) index with the flag (-a bwtsw), and reads were mapped to that reference genome using STAR aligner (v2.7.0d).^67^ The resulting SAM files were sorted, indexed, and converted to BAM files using SAMtools (v1.16). Only uniquely mapped non-duplicated reads were used for SNP calling according to the best practices pipeline of GATK (v3.7).^68^ Duplicated reads in alignment BAM files were marked using Picard Tools (http://picard.sourceforge.net). SNPs were called based on a minimum phred-scaled confidence threshold of 20 (-stand_call_conf > 20) using the GATK tool HaplotypeCaller and then filtered using the GATK tool VariantFiltration with the following requirements: Fisher strand value (FS) < 30.0 and quality by depth value (QD) > 2.0.^68^ For GWAS and eQTL analysis, SNPs having a high missingness rate (> 20%) or low MAF (< 0.05) were removed using VCFtools (v0.1.16) with the parameters (--remove-indels, --maf 0.05, --max-maf 0.95, --max-missing 0.8).^66^ Missing genotypes were imputed using Beagle (v3.1.1) with the following parameters (window = 50000, overlap = 5000, ibd = True).^69^ This process identified 1.19 million autosomal SNPs, output in a variant call format (VCF) file.

### RNA-seq library construction and transcriptome sequencing

For RNA profiling, 20-DPA fibers were harvested from 12:00 to 13:00. The aim was to collect samples in the shortest amount of time possible to minimize the effects of physiological changes. Harvested ovules were frozen with liquid nitrogen for RNA extraction. Total RNA was extracted by the Trizol (Invitrogen) method according to the manufacturer’s instructions, and RNA quality was verified with an Agilent 2100 Bioanalyzer (Agilent). Transcriptome libraries were constructed according to the standard Illumina RNA-seq protocol (Illumina, Inc., San Diego, CA, USA, Cat# RS-100-0801). RNA and DNA sequences were generated as 150 bp paired-end reads from libraries having inserts of 350 bp.

### RNA-seq mapping and analysis

For each genotype, mRNA-seq libraries were constructed with two biological replications and were paired-end sequenced for 126 cycles. RNA-seq reads were aligned to the reference genome (TM-1) using Hisat2 (v2.1.0).^70^ Transcript abundance was quantified with StringTie (v1.3.3b)^70^ and normalized to fragments per kilobase of transcript per million reads (FPKM). Only genes having an FPKM ≥ 1 in ≥ 5% sample were included.

### LncRNA analysis and prediction

To examine the expression of non-coding sequences, we performed population-level transcript assembly of lncRNAs. An average of 24.34 million reads was obtained from each library. Clean reads (150 bp paired-end) were aligned to the TM-1 v2.1 reference genome using Hisat2 (v2.1.0) with parameter (--dta).^71^ Mapped reads in each library were subsequently passed to StringTie (v1.3.3b) for transcript assembly^71^ using annotated TM-1 transcripts^34^ as the reference transcriptome; the assembled transcripts were combined into a unified set using cuffmerge with parameter (-c 3).^70^ Transcripts of less than 200 nt were discarded. Using Cuffcompare (v2.2.1), transcripts were given a class code of “u”, respectively, representing intergenic sequences, antisense sequences of known genes, and intronic sequences. The Coding Potential Calculator2 (CPC2) (v0.1)^72^ was used to calculate the coding potential of transcripts of each given class (“u”) with default parameters. All transcripts with CPC scores > 0 were discarded. The remaining transcripts were subjected to pfam_scan to exclude those containing known protein domains (cutoff < 0.001).^73^ The transcripts left after that step were considered candidate lncRNAs. To reduce isoform complexity, only the longest transcript of each locus was used for further analysis.

### eQTM analysis

To study the relationship of DNA methylation variation with gene expression, we examined SMPs located within 1 Mb of the midpoint of each gene. We treated methylation levels as marked and the expression of individual genes as the phenotype and assumed that each phenotype can be modeled as *y* = 1 using a linear mixed model approach by fastQTL (v7, https://github.com/francois-a/fastqtl).^40^ Gene expression was quantile-normalized to the standard normal distribution *N* (0,1) as phenotype.

### *cis*-meQTLs analysis

To study the relationship of genetic variants with DNA methylation, we extracted SNPs located within 1 Mb of the midpoint of each SMP (MAF > 0.05). We treated the methylation levels at individual DNA methylation sites as phenotypes and assumed that each phenotype could be modeled as y = 1 using a linear mixed model approach by fastQTL (v7).^40^ To control bias across samples, PCA was performed. The analysis incorporated three PCs for population stratification and two additional PCs as unknown confounders. The methylation level of each locus was quantile-normalized to the standard normal distribution *N* (0,1) as the phenotype. The fastQTL (v7) was used to perform a permutation-based meQTL search for each DNA methylation site, calculating the empirical *P* value for the SNP with the strongest genetic effect.^40^

### *trans*-meQTLs analysis

meQTL was performed over a total of 1.19 million SNPs (MAF > 5% and missing rate < 20%). Population structure was calculated using GCTA (v1.92.1) with the parameters (--make-grm --pca).^74^ The first three genotyping principal components (PCs) and kinship matrix were employed as covariates to control false-positive associations. Genotype files were transposed using plink (v1.9) with the parameters (--bfile –recode12 –output-missing-genotype0 –transpose --out).^75^ Kinship matrices were obtained using the emmax-kin function of EMMAX (v07Mar2010) with parameters (-v -d 10).^44^ The DNA methylation levels of each site was used to be molecular phenotype. meQTL mapping was carried out using EMMAX with a mixed linear model and parameters (-v -d 10 -t -o -k -c).^44^ The effective number of independent SNPs was calculated using the Genetic Type I Error Calculator (GEC, v1.0),^76^ and significant SNPs were identified using the threshold of *P* < 2.18 × 10^‒6^ .^76^ To reduce meQTL redundancy, we conducted LD analysis for the associated SNPs. Lead SNPs within a given LD block (*R*^*2*^ > 0.1) associated with a trait were merged into one meQTL using plink (v1.90) with parameters (-r2 -l -window 99999).^75^ The meQTLs were then further classified as *cis-*meQTLs or *trans-*meQTLs based on the distance between the marker SNP and the associated SMP (threshold: 1 Mb).

### eQTLs

The analysis included 207 accessions for which genotype and gene expression data were available. GWAS was performed over a total of 1.19 million SNPs (MAF > 5% and missing rate < 20%). Population structure was calculated using GCTA (v1.92.1) with the parameters (--make-grm --pca).^74^ Only genes having FPKM > 1 in more than 5% of accessions were defined as expressed for eQTL mapping. The expression of each gene was normalized using QQ-normal in R as is commonly done in QTL studies.^77^ Ultimately, a dataset comprising 42,858 PCGs and 6779 lncRNAs was obtained and used to conduct downstream analyses. The first three genotyping PCs and kinship matrix were employed as covariates to control false-positive associations. Genotype files were transposed using plink (v1.9) with the parameters (--bfile –recode12 –output-missing-genotype0 –transpose --out).^75^ Kinship matrices were obtained using the emmax-kin function of EMMAX (v07Mar2010) with parameters (-v -d 10).^44^ eQTL mapping was carried out using EMMAX (v 07Mar2010) with a mixed linear model and parameters (-v -d 10 -t -o -k -c).^44^ The effective number of independent SNPs was calculated using the Genetic Type I Error Calculator (GEC, v1.0),^76^ and significant SNPs were identified using the threshold of *P* < 2.18 × 10^‒6^ suggested by GEC (v1.0).^76^ To reduce eQTL redundancy, we conducted LD analysis for the associated SNPs. Lead SNPs within a given LD block (*R*^*2*^ > 0.1) associated with a trait were merged into one eQTL using plink (v1.90) with parameters (-r2 -l -window 99999).^75^ The eQTLs were then further classified as *cis-*eQTLs or *trans-*eQTLs based on the distance between the marker SNP and the transcription start or end sites of associated genes (threshold: 1 Mb).

### EWAS

A large-scale EWAS was carried out using SMP with MAF > 0.05. Mapping was carried out using EMMAX with a mixed linear model and parameters (-v -d 10 -t -o -k -c).^44^ The effective number of independent SMPs was calculated using the Genetic Type I Error Calculator (GEC, v1.0),^76^ and significant SMPs were identified using the threshold suggested by GEC (v1.0).^76^

### Plant materials, vector construction, and genetic transformation

The cotton used in this study was *G. hirsutum* cv 668. Transgenic lines were planted in a greenhouse at Zhejiang University, Hangzhou, China. The greenhouse was kept at 28 °C with a 14-h light/10-h dark photoperiod. The CRISPR-Cas9-mediated gene editing vector was constructed as described previously.^78^ Transgenic plants were created by *Agrobacterium*-mediated transformation. Mutation analysis of *CIPK10* (*GH_A03G0334*) CRISPR-Cas9 transgenic plants utilized the Hi-TOM method as described previously.^79^ Plants found to carry *CIPK10* mutations were chosen for phenotypic analysis.

### Phenotype prediction

Two representative algorithms, G2Pdeep^80^ and the linear models Genomic Best Linear Unbiased Prediction (GBLUP) method^81^ were employed for each trait prediction. The SNPs used in trait prediction were sourced from eQTL analysis, while the SMP used in trait prediction were sourced from eQTM analysis. In order to prevent data leakage, loci identified in EWAS and GWAS were excluded from the model construction process. The predictive performance of the models was compared using the PCC between the predicted (Yˆ)and the true trait value (Y).

### Functional DNA methylation locus prediction using a deep neural network

A total of 2336 CG loci from 2423 CG-eQTMs were considered to be functional DNA methylation sites, i.e., positive samples; matching 2336 DNA methylation sites were randomly selected as negative samples. For each sample, a DNA sequence of 8192 bp centered at the CG methylation site is extracted and one-hot-encoded (A = (1,0,0,0), C = (0,1,0,0), G = (0,0,1,0), T = (0,0,0,1), N = (0,0,0,0)) to serve as model input. Since the Enformer model^52^ was trained on a large amount of human genomic data, we used its core as our backbone, i.e., the convolution part with 7 convolution-pool blocks and the transformer part with 11 transformer encoding layers. The convolution part down-samples the input sequence by 128 and extracts local sequence features, while the transformer part aggregates long-range global features. This backbone transforms inputs into features of shape 64 × 3072. The middle features of shape 8 × 3072 are then flattened and fed to the output layer, which is a fully connected layer and predicts whether the site in question is a functional DNA methylation site.

Prediction experiments were implemented using the PyTorch framework^82^ with four NVIDIA Tesla P100 GPUs. The Adam optimizer was applied with an initial learning rate of 1 × 10^‒4^ and weight decay of 1 × 10^‒8^. Each mini-batch contained 64 samples. In each training period, we trained the deep model up to ten epochs. All experiments used binary cross-entropy as the loss function, and 10-fold cross-validation was applied to evaluate the results.

## Supplementary information

Supplementary information, Fig. S1. Assessment of data quality generated in this study.

Supplementary information, Fig. S2. Circos plot showing single methylation polymorphisms in our cotton population.

Supplementary information, Fig. S3. Bar plot showed the distribution of methylated levels for CG, CHG, and CHH loci in five randomly sampled accessions.

Supplementary information, Fig. S4. Phylogenetic tree constructed based on the SMPs.

Supplementary information, Fig. S5. The genomic distribution of common SMP.

Supplementary information, Fig. S6. The cis-meQTLs were enriched in the gene- rich region on chromosome.

Supplementary information, Fig. S7. The impact of DNA methylation on gene expression.

Supplementary information, Fig. S8. The expression pattern of genes encoding critical proteins in DNA methylation establishment.

Supplementary information, Fig. S9. Identification and characterization of EWAS loci.

Supplementary information, Fig. S10. The accumulated effects of elite epi-alleles.

Supplementary information, Fig. S11. Prediction of trait based on utilizing a combination of functional SNPs and SMPs.

Supplementary information, Fig. S12. Sequence analysis of CIPK10 mutants KO1 and KO2 at the target sites.

Supplementary information, Table S1. Summary of bisulfite sequencing for fiber tissues of 207 cotton accessions.

Supplementary information, Table S2. Summary of RNA-Seq for fiber tissues of 207 cotton accessions.

Supplementary information, Table S3: Annotation of SMP and SNP in gene-related regions.

Supplementary information, Table S4: Identification and characterization of genes regulated by DNA methylation.

Supplementary information, Table S5: GO enrichment analysis of eQTM genes.

Supplementary information, Table S6: Identification and characterization of genes regulated by genetic and epigenetic variation.

Supplementary information, Table S7: Genes in the DNA methylation pathway

Supplementary information, Table S8: Identification and characterization of epi-alleles associated with agronomic traits

Supplementary information, Table S9: Identification of genes related to fiber traits by integrating multiple omics

## Data Availability

All RNA-seq and BS-seq have been deposited in the NCBI Short Read Archive (https://www.ncbi.nlm.nih.gov/sra) under respective Bioproject PRJNA1146873. Sample IDs and metadata can be found in Supplementary information, Tables S1 and S2.
