# Supplementary material for: Population-wide DNA methylation polymorphisms at single-nucleotide resolution in 207 cotton accessions reveal epigenomic contributions to complex traits
Source: Cell Res. 2024 Oct 17;34(12):859–72. doi: 10.1038/s41422-024-01027-x (PMC11615300; doi:10.1038/s41422-024-01027-x)
Supplement: Supplementary file 1 — Supplementary information, Fig. S1. Assessment of data quality generated in this study. [file 41422_2024_1027_MOESM1_ESM.pdf]

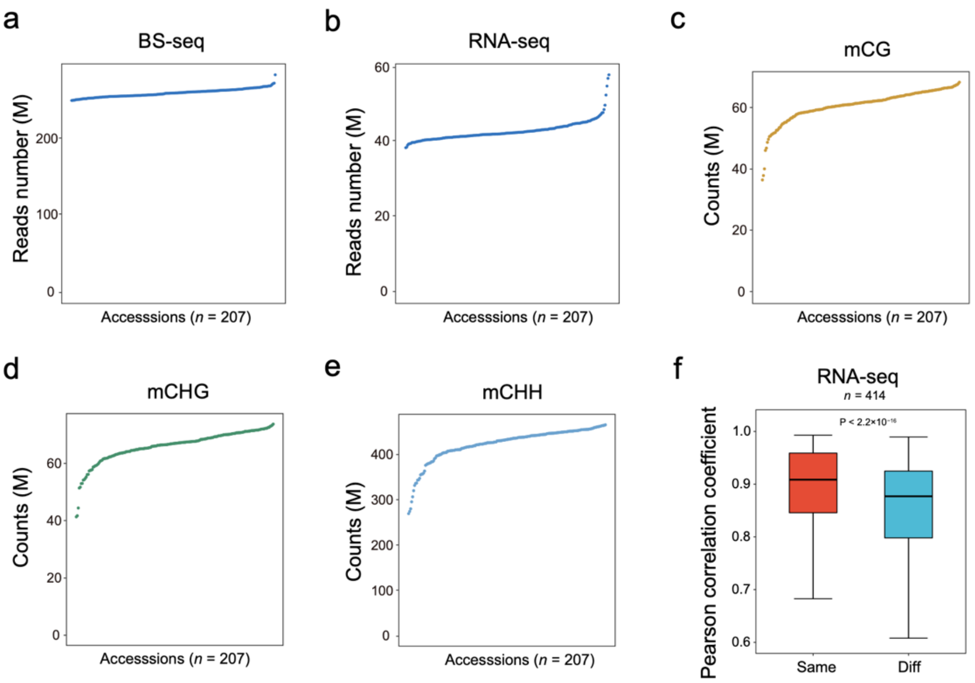

Supplementary information, Fig. S1. Assessment of data quality generated in this study. a-b, A dot plot was utilized to present the read counts of BS-seq (a) and RNA-seq (b) for different accessions. The x-axis denotes the various accessions, while the y-axis shows the read counts. **c-e,** The dot plot depicted the quantified (c) CG loci, (d) CHG loci, and (e) CHH loci with each point representing an individual accession. **f,** The boxplot illustrated the Pearson correlation coefficient (*PCC*) of gene expression quantifications among samples. It compares replicates of the same accessions (Same) and randomly selected samples from different accessions (Diff). The quantifications were normalized to FPKM before calculating the pairwise *PCC*.
