# Supplementary material for: Population-wide DNA methylation polymorphisms at single-nucleotide resolution in 207 cotton accessions reveal epigenomic contributions to complex traits
Source: Cell Res. 2024 Oct 17;34(12):859–72. doi: 10.1038/s41422-024-01027-x (PMC11615300; doi:10.1038/s41422-024-01027-x)
Supplement: Supplementary file 2 — Supplementary information, Fig. S2. Circos plot showing single methylation polymorphisms in our cotton population. [file 41422_2024_1027_MOESM2_ESM.pdf]

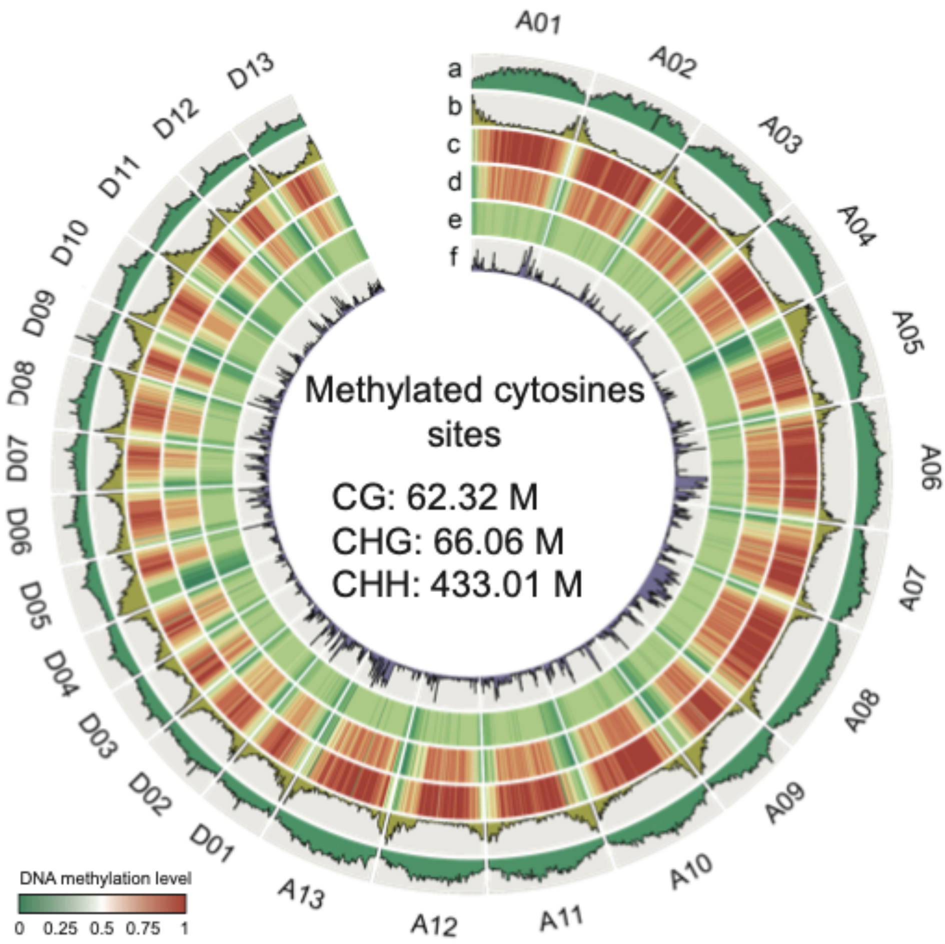

Supplementary information, Fig. S2. Circos plot showing single methylation polymorphisms in our cotton population. The outer track represents the 26 chromosomes in the *G. hirsutum* genome (A01-A13 of the A subgenome and D01-D13 of the D subgenome). Circles from a to f respectively represent (a)TE content; (b) number of protein coding genes (PCGs). (c) weighted DNA methylation levels at CG, (d)CHG, and (e) CHH sites; and the (f) distribution of SNPs. Data for each chromosome were analyzed in 1-Mb windows. Weighted methylation levels using running medians in 1-Mb windows are shown from low (0, green) to high (1, blue).
