# Supplementary material for: Population-wide DNA methylation polymorphisms at single-nucleotide resolution in 207 cotton accessions reveal epigenomic contributions to complex traits
Source: Cell Res. 2024 Oct 17;34(12):859–72. doi: 10.1038/s41422-024-01027-x (PMC11615300; doi:10.1038/s41422-024-01027-x)
Supplement: Supplementary file 3 — Supplementary information, Fig. S3. Bar plot showed the distribution of methylated levels for CG, CHG, and CHH loci in five randomly sampled accessions. [file 41422_2024_1027_MOESM3_ESM.pdf]

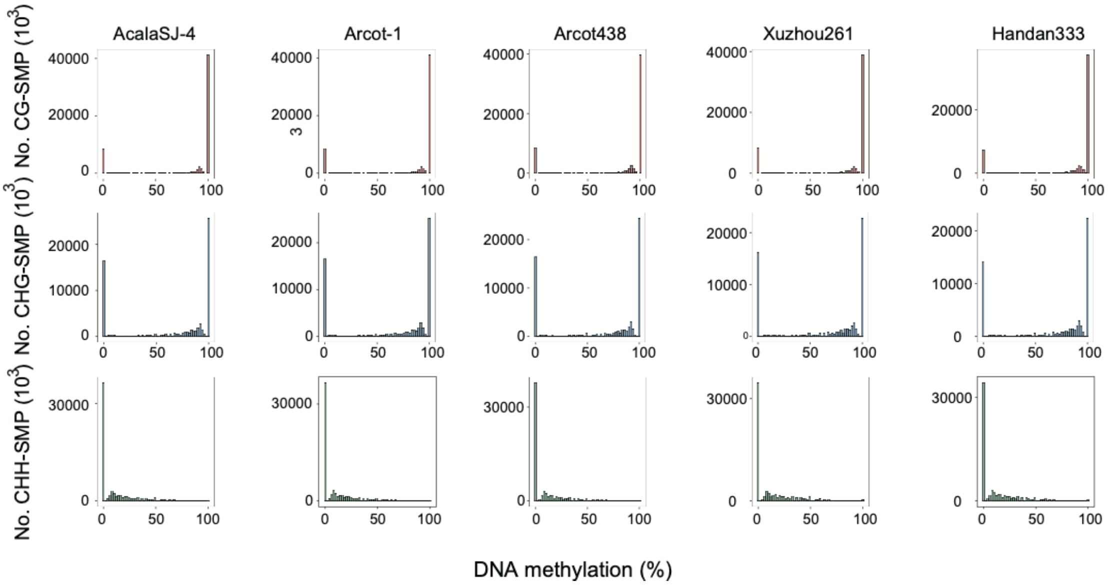


**Supplementary information, Fig. S3. Bar plot showed the distribution of methylated levels for CG, CHG, and CHH loci in five randomly sampled accessions.** The accessions, named acalaSJ-4, Arcot-1, Arcot438, Xuzhou261, and Handan333, are arranged from left to right. The top to bottom order indicates the distribution of DNA methylation levels of CG, CHG, and CHH, respectively.
