# Supplementary figures and images for: Population-wide DNA methylation polymorphisms at single-nucleotide resolution in 207 cotton accessions reveal epigenomic contributions to complex traits
Source: Cell Res. 2024 Oct 17;34(12):859–72. doi: 10.1038/s41422-024-01027-x (PMC11615300; doi:10.1038/s41422-024-01027-x)

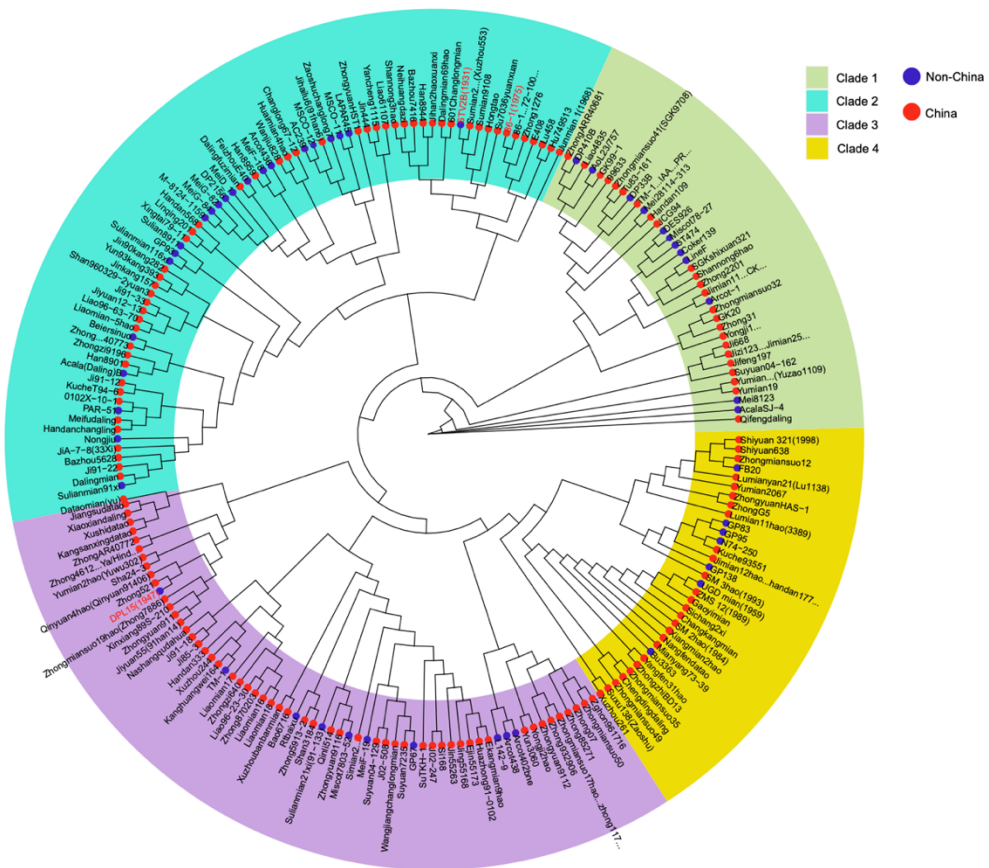

**Supplementary information, Fig. S4. Phylogenetic tree constructed based on the SMPs.**

Supplement: Supplementary file 4 — Supplementary information, Fig. S4. Phylogenetic tree constructed based on the SMPs. [file 41422_2024_1027_MOESM4_ESM.pdf]
