# Supplementary material for: Population-wide DNA methylation polymorphisms at single-nucleotide resolution in 207 cotton accessions reveal epigenomic contributions to complex traits
Source: Cell Res. 2024 Oct 17;34(12):859–72. doi: 10.1038/s41422-024-01027-x (PMC11615300; doi:10.1038/s41422-024-01027-x)
Supplement: Supplementary file 5 — Supplementary information, Fig. S5. The genomic distribution of common SMP. [file 41422_2024_1027_MOESM5_ESM.pdf]

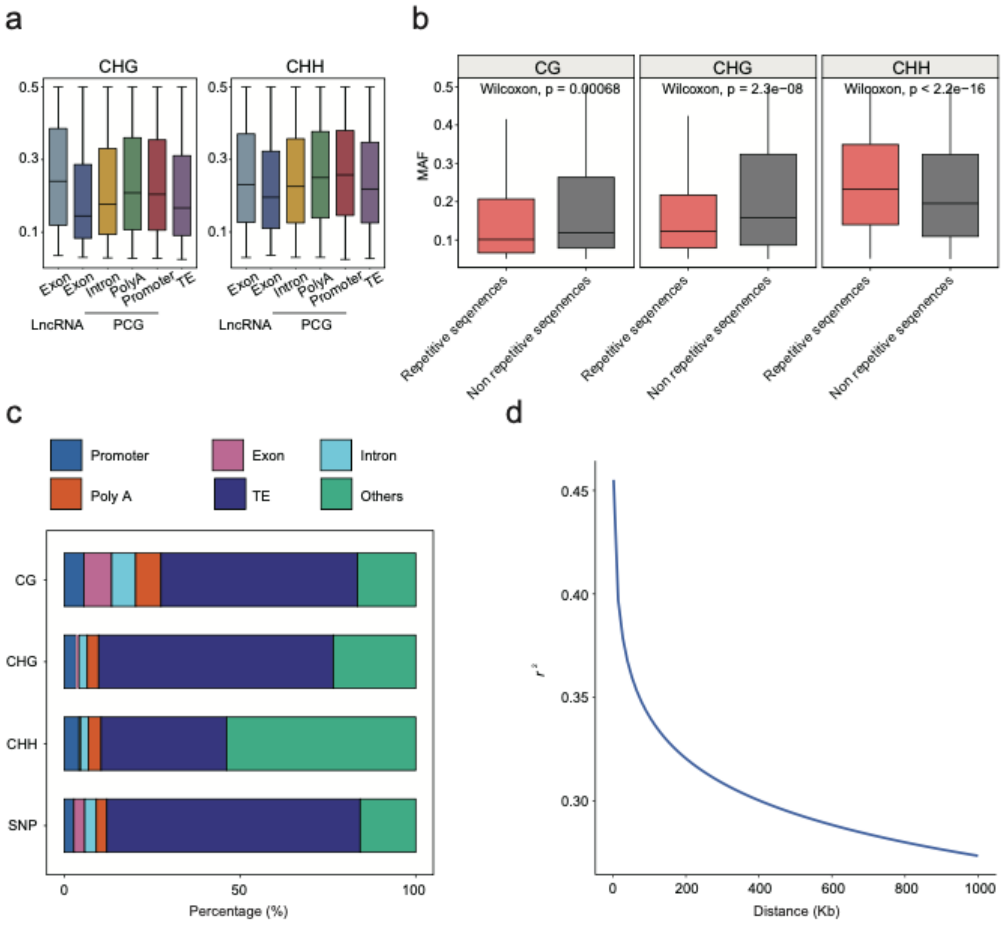


**Supplementary information, Fig. S5. The genomic distribution of common SMP.** **a,** The Boxplot showed the distribution of minor allele frequency (MAF) of CHG-SMP and CHH-SMP across different genomic features. The context of CHG, and CHH were arranged from left to right. **b,** The minor allele frequency (MAF) of single nucleotide polymorphisms (SMPs) in both repetitive and non-repetitive sequences within gene exons. **c,** The source of common SMP (MAF $\geq$ 0.05) across different genomic features. The common SNP were selected as control. **d,** linkage disequilibrium decay of SNPs.
