# Supplementary material for: Population-wide DNA methylation polymorphisms at single-nucleotide resolution in 207 cotton accessions reveal epigenomic contributions to complex traits
Source: Cell Res. 2024 Oct 17;34(12):859–72. doi: 10.1038/s41422-024-01027-x (PMC11615300; doi:10.1038/s41422-024-01027-x)
Supplement: Supplementary file 6 — Supplementary information, Fig. S6. The cis-meQTLs were enriched in the gene- rich region on chromosome. [file 41422_2024_1027_MOESM6_ESM.pdf]

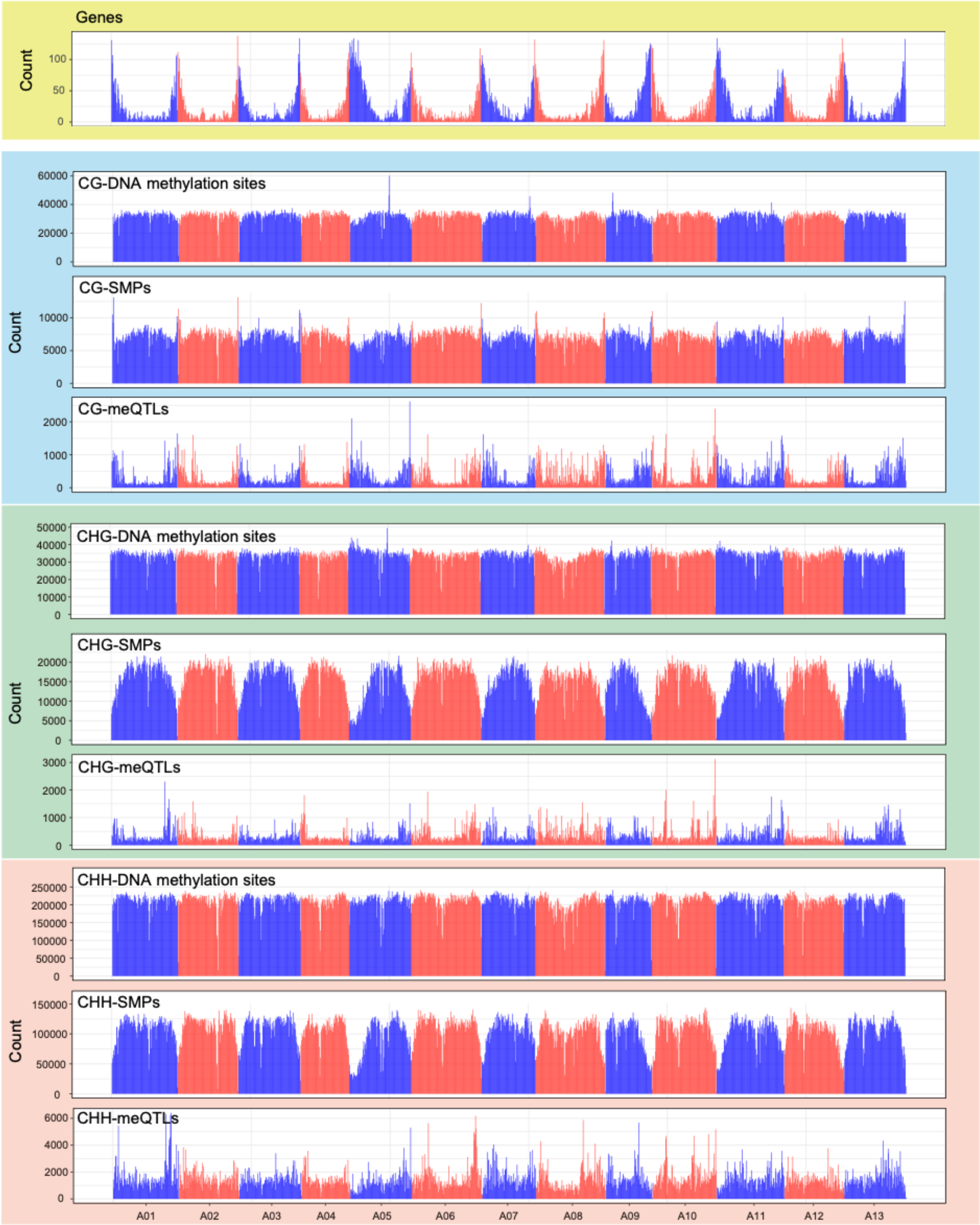

Supplementary information, Fig. S6. The *cis*-meQTLs were enriched in the gene-rich region on chromosome. Distribution of meQTL across the genome. From top to bottom, is counts of genes, counts of CG DNA methylation sites, CG-SMP, CG-meQTL, counts of CHG DNA methylation sites, CHG-SMP, CHG-meQTL, counts of CHH DNA methylation sites, CHH-SMP, CHH-meQTL, respectively. Each type of data was measured in 1-Mb windows.
