# Supplementary material for: Population-wide DNA methylation polymorphisms at single-nucleotide resolution in 207 cotton accessions reveal epigenomic contributions to complex traits
Source: Cell Res. 2024 Oct 17;34(12):859–72. doi: 10.1038/s41422-024-01027-x (PMC11615300; doi:10.1038/s41422-024-01027-x)
Supplement: Supplementary file 7 — Supplementary information, Fig. S7. The impact of DNA methylation on gene expression. [file 41422_2024_1027_MOESM7_ESM.pdf]

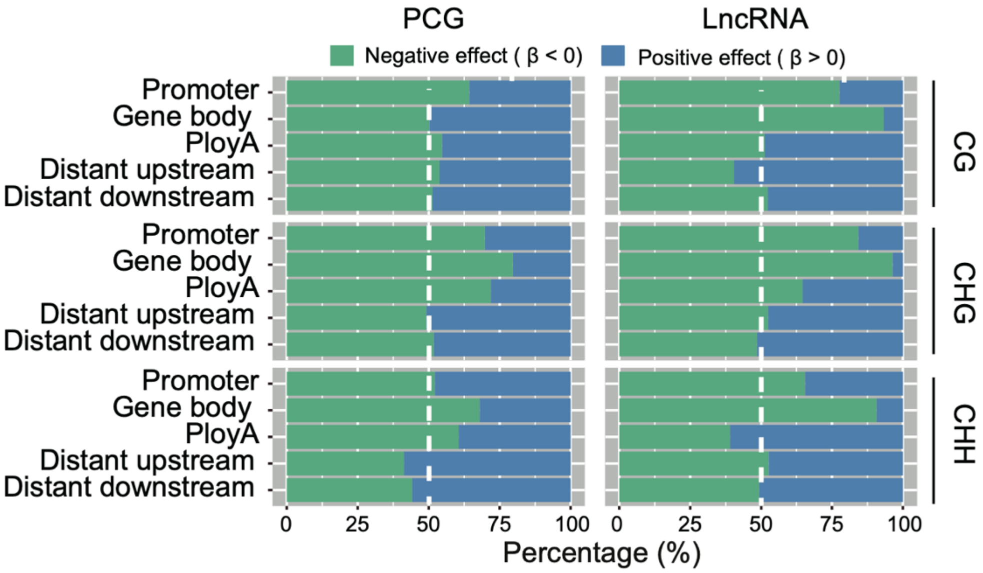

Supplementary information, Fig. S7. The impact of DNA methylation on gene expression. The effect of *cis*-eQTM across different genomic feature (Promoter, Gene body, Poly (A), distant upstream (> -2000 bp), and distant downstream (> 2000 bp). The effect of each *cis*-eQTM were grouped into negative (beta < 0) and positive (beta > 0).
