# Supplementary material for: Population-wide DNA methylation polymorphisms at single-nucleotide resolution in 207 cotton accessions reveal epigenomic contributions to complex traits
Source: Cell Res. 2024 Oct 17;34(12):859–72. doi: 10.1038/s41422-024-01027-x (PMC11615300; doi:10.1038/s41422-024-01027-x)
Supplement: Supplementary file 8 — Supplementary information, Fig. S8. The expression pattern of genes encoding critical proteins in DNA methylation establishment. [file 41422_2024_1027_MOESM8_ESM.pdf]

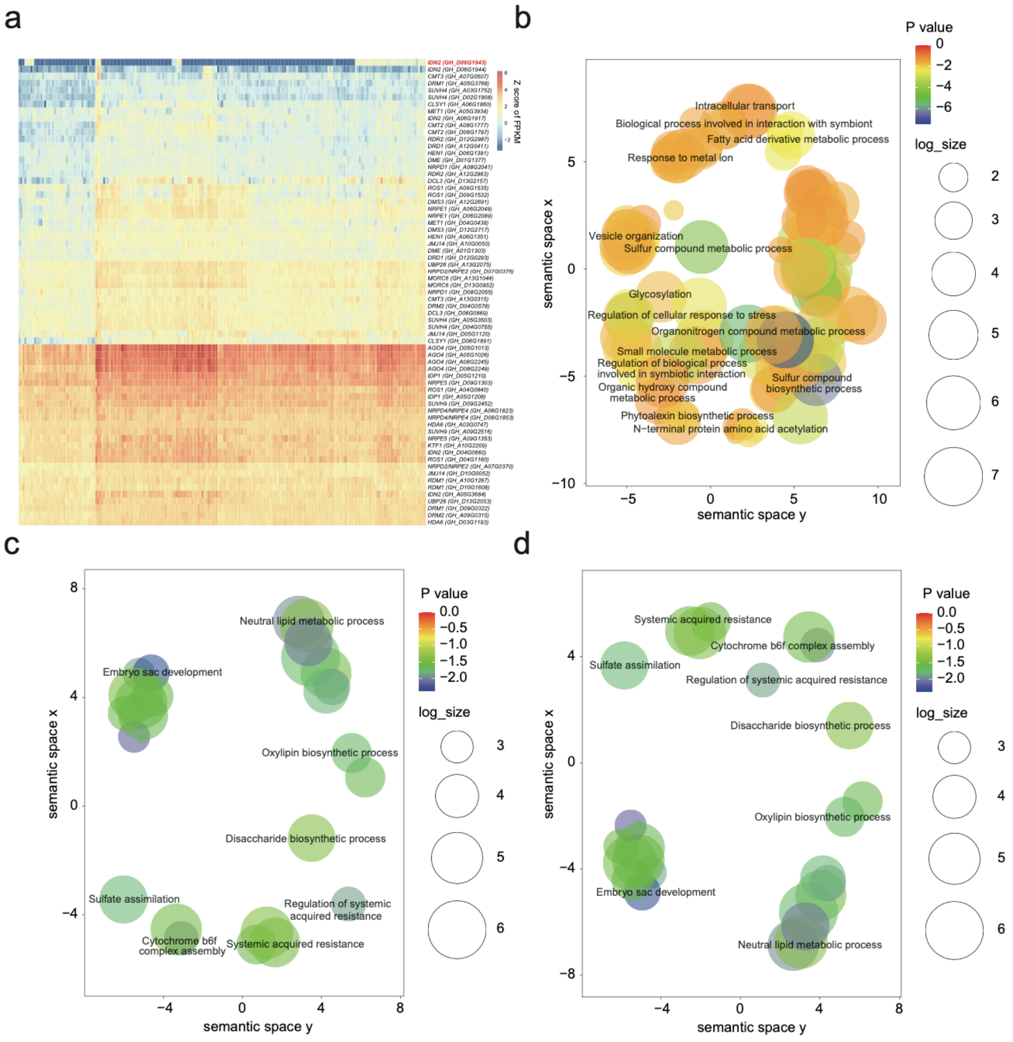

Supplementary information, Fig. S8. The expression pattern of genes encoding critical proteins in DNA methylation establishment. a, The heatmap visually represents the levels of gene expression (Z score of FPKM) for DNA methyltransferase, with the eGenes of *cis*-eQTL highlighted in red. **b,** Gene Ontology (GO) analysis of co-regulated genes. **c, d,** Gene Ontology (GO) analysis of eQTL-specific(c) and eQTM-specific genes(d).
