# Supplementary material for: Population-wide DNA methylation polymorphisms at single-nucleotide resolution in 207 cotton accessions reveal epigenomic contributions to complex traits
Source: Cell Res. 2024 Oct 17;34(12):859–72. doi: 10.1038/s41422-024-01027-x (PMC11615300; doi:10.1038/s41422-024-01027-x)
Supplement: Supplementary file 9 — Supplementary information, Fig. S9. Identification and characterization of EWAS loci. [file 41422_2024_1027_MOESM9_ESM.pdf]

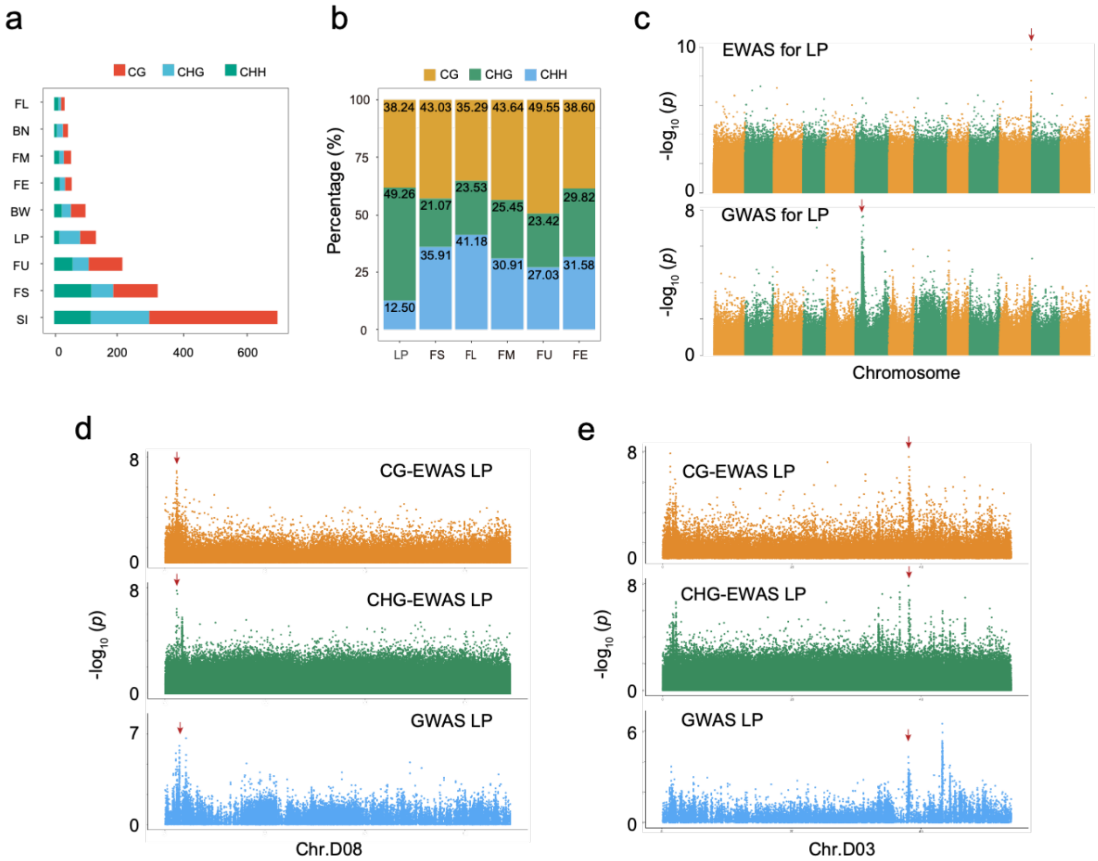

Supplementary information, Fig. S9. Identification and characterization of EWAS loci. a, bar plot displayed the number of CG-EWAS (in red), CHG-EWAS (in blue), and CHH-EWAS (in green) loci for different traits. **b,** A bar plot showed the percentage of CG-EWAS loci (yellow), CHG-EWAS loci (green) and CHH-EWAS loci (blue), respectively. **c,** Combined Manhattan plots for GWAS and EWAS for lint percentage (LP) trait. **d-e,** Combined Manhattan plots for CG-EWAS and CHG-EWAS for LP trait in Chr. D08 (d) and Chr. D03 (e)
