# Supplementary material for: Population-wide DNA methylation polymorphisms at single-nucleotide resolution in 207 cotton accessions reveal epigenomic contributions to complex traits
Source: Cell Res. 2024 Oct 17;34(12):859–72. doi: 10.1038/s41422-024-01027-x (PMC11615300; doi:10.1038/s41422-024-01027-x)
Supplement: Supplementary file 10 — Supplementary information, Fig. S10. The accumulated effects of elite epi-alleles. [file 41422_2024_1027_MOESM10_ESM.pdf]

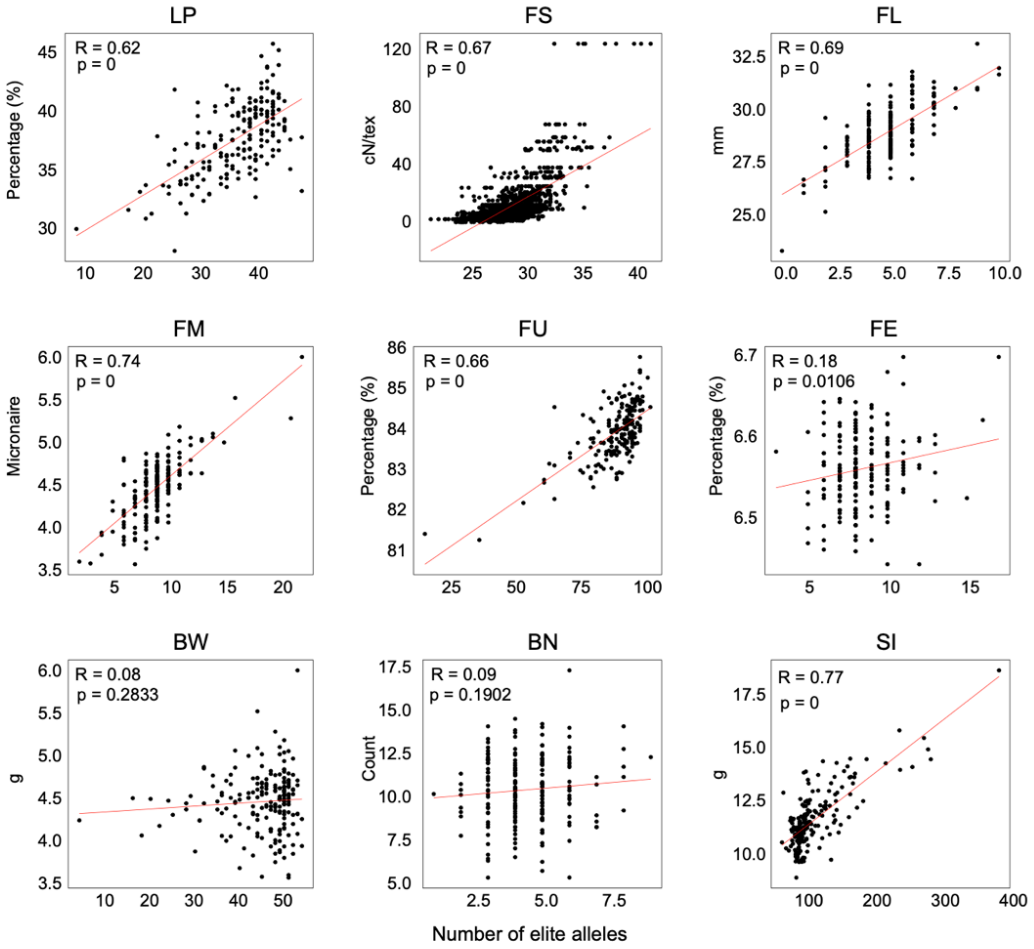

Supplementary information, Fig. S10. The accumulated effects of elite epi-alleles. The dot plot displayed the accumulated effects of negative EWAS loci on various traits in different environments. The *x*-axis represents the number of favorable alleles, while the *y*-axis represents the corresponding trait value.
