# Supplementary material for: Population-wide DNA methylation polymorphisms at single-nucleotide resolution in 207 cotton accessions reveal epigenomic contributions to complex traits
Source: Cell Res. 2024 Oct 17;34(12):859–72. doi: 10.1038/s41422-024-01027-x (PMC11615300; doi:10.1038/s41422-024-01027-x)
Supplement: Supplementary file 11 — Supplementary information, Fig. S11. Prediction of trait based on utilizing a combination of functional SNPs and SMPs. [file 41422_2024_1027_MOESM11_ESM.pdf]

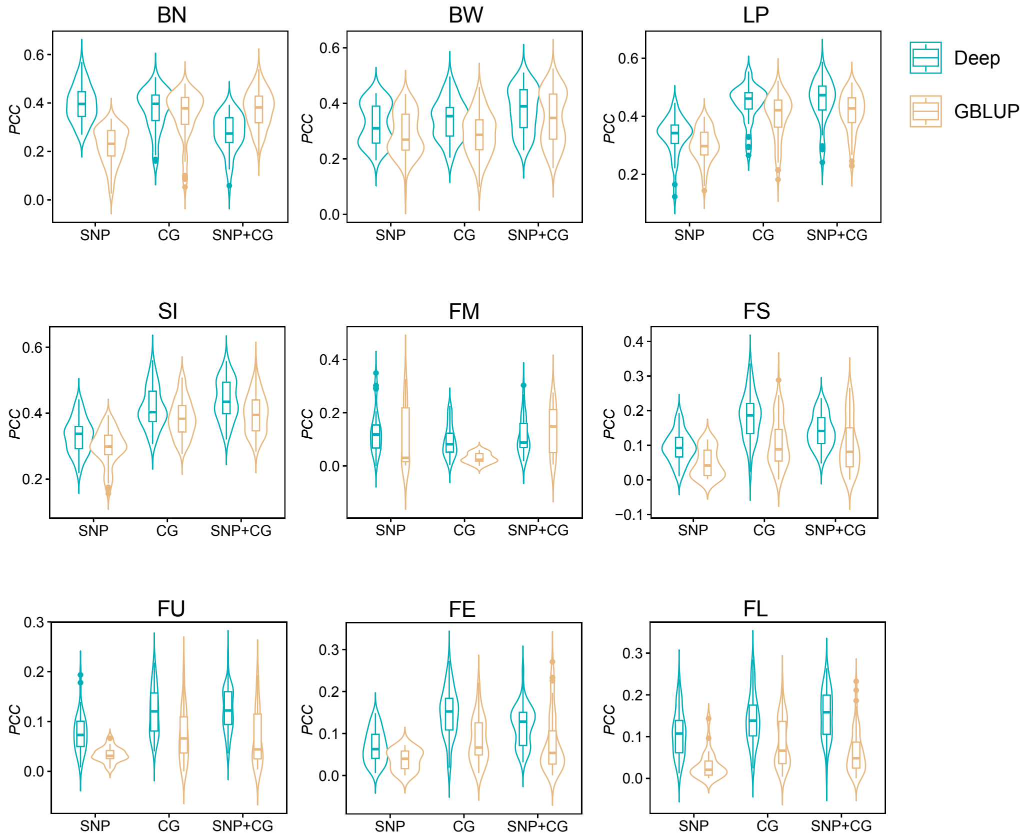

Supplementary information, Fig. S11. Prediction of trait based on utilizing a combination of functional SNPs and SMPs. The *y*-axis plots the *PCC*.
