# Supplementary material for: Population-wide DNA methylation polymorphisms at single-nucleotide resolution in 207 cotton accessions reveal epigenomic contributions to complex traits
Source: Cell Res. 2024 Oct 17;34(12):859–72. doi: 10.1038/s41422-024-01027-x (PMC11615300; doi:10.1038/s41422-024-01027-x)
Supplement: Supplementary file 12 — Supplementary information, Fig. S12. Sequence analysis of CIPK10 mutants KO1 and KO2 at the target sites. [file 41422_2024_1027_MOESM12_ESM.pdf]

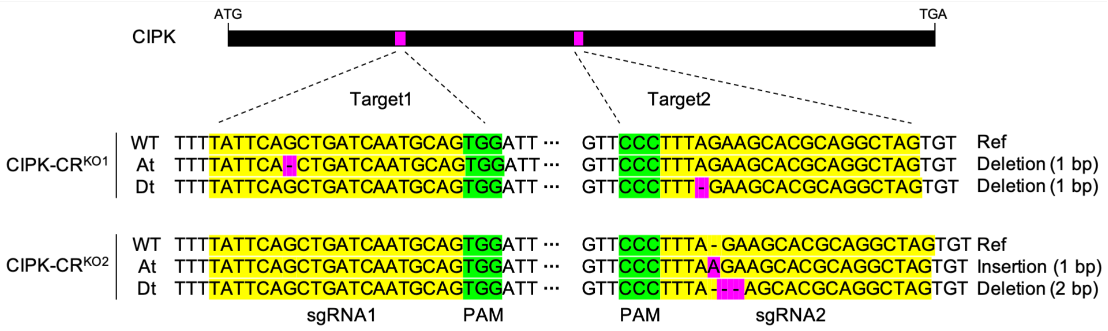

Supplementary information, Fig. S12. Sequence analysis of *CIPK10* mutants KO1 and KO2 at the target sites. The PAM motif is marked in green and *CIPK_A$_T$* and *CIPK10_D$_T$* can be distinguished by the SNP. The purple color represents deletion compared with WT.
